# Region-selective control of the thalamic reticular nucleus via cortical layer 5 pyramidal cells

**DOI:** 10.1038/s41593-022-01217-z

**Published:** 2022-12-22

**Authors:** Nóra Hádinger, Emília Bősz, Boglárka Tóth, Gil Vantomme, Anita Lüthi, László Acsády

**Affiliations:** 1 Laboratory of Thalamus Research, Institute of Experimental Medicine, Budapest, Hungary; 2 János Szentágothai Doctoral School of Neurosciences, Semmelweis University, Budapest, Hungary; 3 Department of Fundamental Neurosciences, University of Lausanne, Lausanne, Switzerland

**Keywords:** Neural circuits, Motor cortex

## Abstract

Corticothalamic pathways, responsible for the top-down control of the thalamus, have a canonical organization such that every cortical region sends output from both layer 6 (L6) and layer 5 (L5) to the thalamus. Here we demonstrate a qualitative, region-specific difference in the organization of mouse corticothalamic pathways. Specifically, L5 pyramidal cells of the frontal cortex, but not other cortical regions, establish monosynaptic connections with the inhibitory thalamic reticular nucleus (TRN). The frontal L5–TRN pathway parallels the L6–TRN projection but has distinct morphological and physiological features. The exact spike output of the L5-contacted TRN cells correlated with the level of cortical synchrony. Optogenetic perturbation of the L5–TRN connection disrupted the tight link between cortical and TRN activity. L5-driven TRN cells innervated thalamic nuclei involved in the control of frontal cortex activity. Our data show that frontal cortex functions require a highly specialized cortical control over intrathalamic inhibitory processes.

## Abstract (web summary)

Hádinger et al. found selective innervation of the thalamic reticular nucleus (TRN) by frontal cortex L5 cells. The spike output of TRN provides a precise readout of the cortical population activity in thalamic regions connected to frontal cortex.

## Main

Thalamocortical circuits underlie the organization of all complex behavior. Every cortical region forms tightly organized, bidirectional connections with the thalamus^1,2^; thus, the thalamus forms an integral part of the cortical network. Thalamocortical circuits involve the excitatory corticothalamic and thalamocortical cells^2^ as well as the GABAergic thalamic reticular nucleus (TRN), which is the main source of intrathalamic inhibition^3^. Corticothalamic circuits are regarded as canonical elements of the forebrain, as no qualitative differences are known to be present between different cortical regions^4^. In contrast to this view, in this study we identified and characterized a region-specific cortico–TRN pathway that arises specifically from the L5 of the frontal cortices.

The TRN is involved in various behavioral processes as sensation^5^, arousal and sleep, including its leading role in the sleep spindle generation^6–8^, selective attention^9,10^, spatial navigation^11^, sensory induced flight responses^12^ and extinction of cued fear conditioning^13^. It is also involved in various pathologies, including attention-deficit hyperactivity disorder, autism^14^, epilepsy^15^ and schizophrenia^16^. Therefore, understanding the regulation of TRN activity and mapping its possible region-specific and behavior-specific aspects are crucial to clarify the basics of thalamocortical functions.

The TRN is at the crossroads of thalamocortical circuits. It receives dense topographic input from the thalamocortical cells and is also contacted by excitatory inputs from corticothalamic cells of all cortical areas^17^. The top-down cortical inputs to TRN are formed by the collaterals of the layer 6 (L6) corticothalamic cells^18^. Consequently, TRN can control the direct effect of the L6 activity on the thalamus in a feed-forward inhibitory manner^19^.

The second corticothalamic pathway involves layer 5 (L5) corticothalamic cells. These corticothalamic inputs are formed by the collaterals of the L5b pyramidal tract (PT) cells and arise from all cortical regions studied so far^4^. PT cells that send axons to the thalamus also innervate many subcortical sites^20^ and, thus, are one of the major pathways through which the cortex can directly impact behavior^21,22^. In contrast to the L6 corticothalamic axons, the available evidence indicates that the L5 axons do not innervate the TRN^23,24^. Accordingly, the impact of the L5 input on the thalamus is not thought to be sculpted by feed-forward inhibition^25^.

Using transgenic mouse lines in which L5 cells were selectively labeled in the neocortex, we demonstrate that L5 PT cells of the frontal cortex—but not other cortical regions—innervate the TRN. This suggests a fundamental spatial heterogeneity in corticothalamic communication. Our anatomical and in vitro electrophysiology data show qualitative differences between L5–TRN and L6–TRN pathways, and our in vivo experiments demonstrate that converging L5 activity on TRN neurons is instrumental to determine the correlation between cortical and TRN activity.

## Results

### L5 innervation of the TRN from the frontal cortex areas

**Fig. 1:**
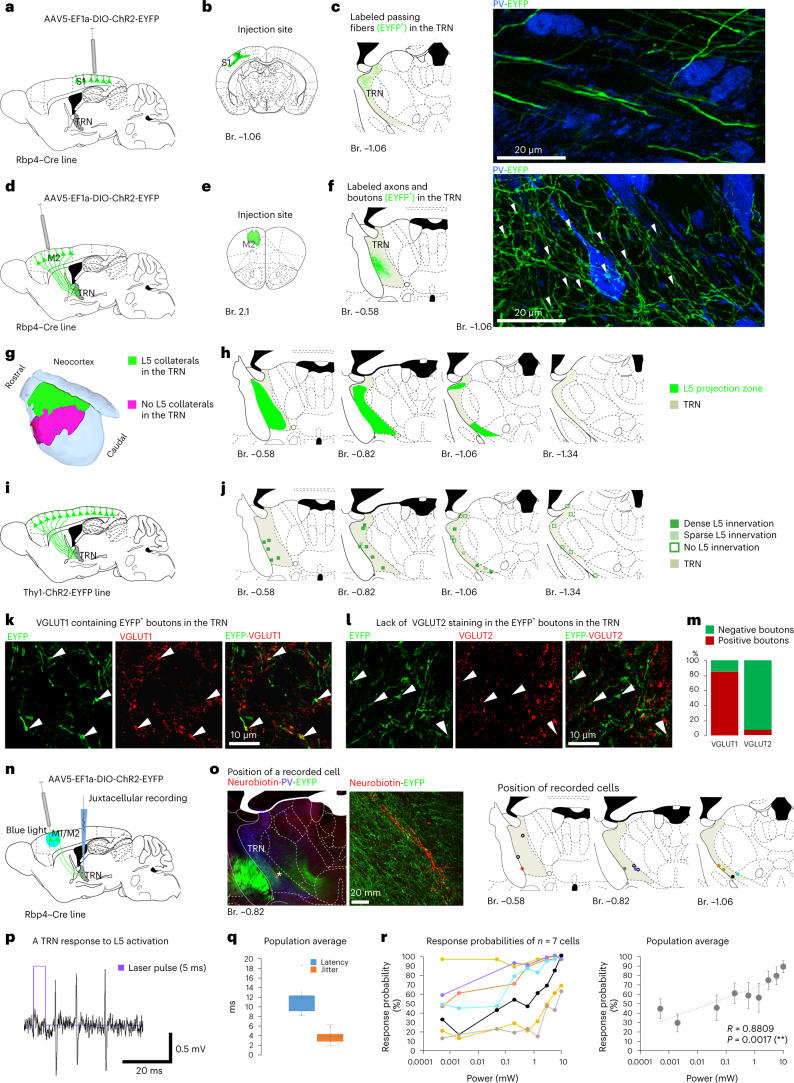
L5 innervation of the TRN from frontal cortex areas. **a**–**c**, Experimental design (**a**), injection site (**b**), schematic figure (**c**, left) and confocal image (**c**, right) of L5 axons (EYFP^+^) in the TRN (PV^+^) after S1 injection. Note the passing L5 axons without boutons in TRN. **d**–**f**, Same after M2 injection. Note the dense meshwork of bouton-bearing (arrowheads) collaterals in TRN. **g**, Cortical regions with (summated areas of *n* = 13 injections in *n* = 13 mice, green) or without (*n* = 5 injections in *n* = 5 mice, magenta) L5 collaterals in TRN. **h**, Distribution of L5–TRN collaterals. Summated area (green) for *n* = 13 cortical injections in *n* = 13 mice. **i**, Experimental design. **j**, Distribution of the L5 boutons in the TRN (*n* = 3 mice) in the Thy1-ChR2-EYFP line. Squares mark the ROIs of confocal images depicting dense (dark green), sparse (light green) or no (empty squares) L5 innervation. Note close correspondence with **h**. **k**, Confocal images of EYFP^+^ terminals in the anterior TRN of the Thy1-Chr2-EYFP mouse (left) and of VGLUT1 immunostaining (middle). Arrowheads: double-positive terminals. **l**, Same as **k**, for VGLUT2. Arrowheads: VGLUT2^−^ terminals. **m**, VGLUT content of the EYFP^+^ boutons (VGLUT1: *n* = 39 boutons in *n* = 3 mice, 84.62% positive; VGLUT2: *n* = 67 boutons in *n* = 3 mice, 7.46% positive). **n**, Experimental design. **o**, Left panels: confocal images of a juxtacellularly labeled TRN neuron surrounded by L5 collaterals (EYFP^+^). TRN is outlined with PV immunostaining in the low-power image. Right panels: position of the recorded neurons (*n* = 11 cells in *n* = 6 mice). Colors match the colors in **r**. **p**, Laser stimulus and TRN response. **q**, Box plots for the latency (11.83 ± 0.84 ms) and jitter (3.96 ± 0.39 ms) of the TRN responses (*n* = 11 cells in *n* = 6 mice; 50 stimuli per cell, 10 mW). **r**, Left: response probabilities of single TRN neurons to L5 stimulation at increasing laser power (50 stimuli per power value). Cells with only 1 activation intensity (black empty squares on **o**) are not involved. Right: population average (*n* = 7 cells in *n* = 4 mice, Pearson correlation: *R* = 0.8809, **P = 0.0017). Error bars depicts average ± s.e.m. Box plots: box shows first to third quartiles; whisker ends indicate minimum and maximum values; x labels the mean. Br., bregma; ROI, region of interest.

**Extended Data Fig. 1:**
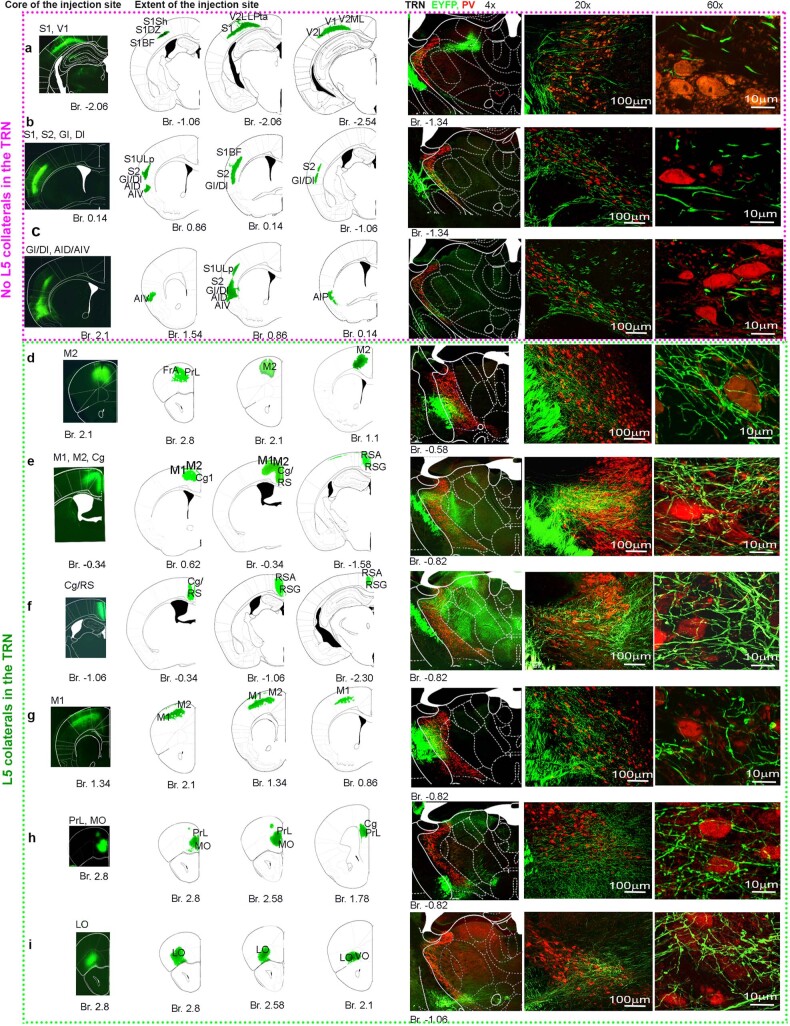
Selective L5 innervation of the TRN from the frontal cortical areas. Related to Fig. 1. (**a-c**): Cortical injections that did not result in L5 collateral labelling in the TRN. L5 cells were virally (AAV5-EF1a-DIO-ChR2_EYFP) labelled in the Rbp4-Cre mouse. Left panels: Fluorescent images of the core of the cortical injection sites. Middle panels: schematic maps showing the extent of the cortical injections. Right panels: Confocal images of the TRN at three different magnifications. In a-c only passing myelinated fibers could be observed in the TRN. Note the lack of dense, synaptic terminal bearing collateral network. L5 fibers are labelled by EYFP (green), boundaries of the TRN are demarcated by PV (red) staining of the somas. (**d-i**): Same as (a-c) for cortical injection sites that resulted L5-collateral labelling in the TRN. Note the dense, synaptic terminal bearing collateral network in the TRN. AID: agranular insular cortex, dorsal part; AIV: agranular insular cortex, ventral part; Br: Bregma; Cg: cingulate cortex; DI: dysgranular insular cortex; GI: granular insular cortex; M1: primary motor cortex; M2: secondary motor cortex; LO: lateral orbital cortex; LPtA: lateral parietal association cortex; MO: medial orbital cortex; Prl: prelimbic cortex; RSA: retrosplenial agranular cortex; RSG: retrosplenial granular cortex ; S1: primary somatosensory cortex; S1BF: primary somatosensory cortex, barrel field; S1DZ: primary somatosensory cortex, dysgranular region; S1Sh: primary somatosensory cortex, shoulder region; S1ULp: primary somatosensory cortex, upper lip region; S2: secondary somatosensory cortex; TRN: thalamic reticular nucleus; V1: primary visual cortex; V2L: secondary visual cortex, lateral area.

**Extended Data Fig. 2:**
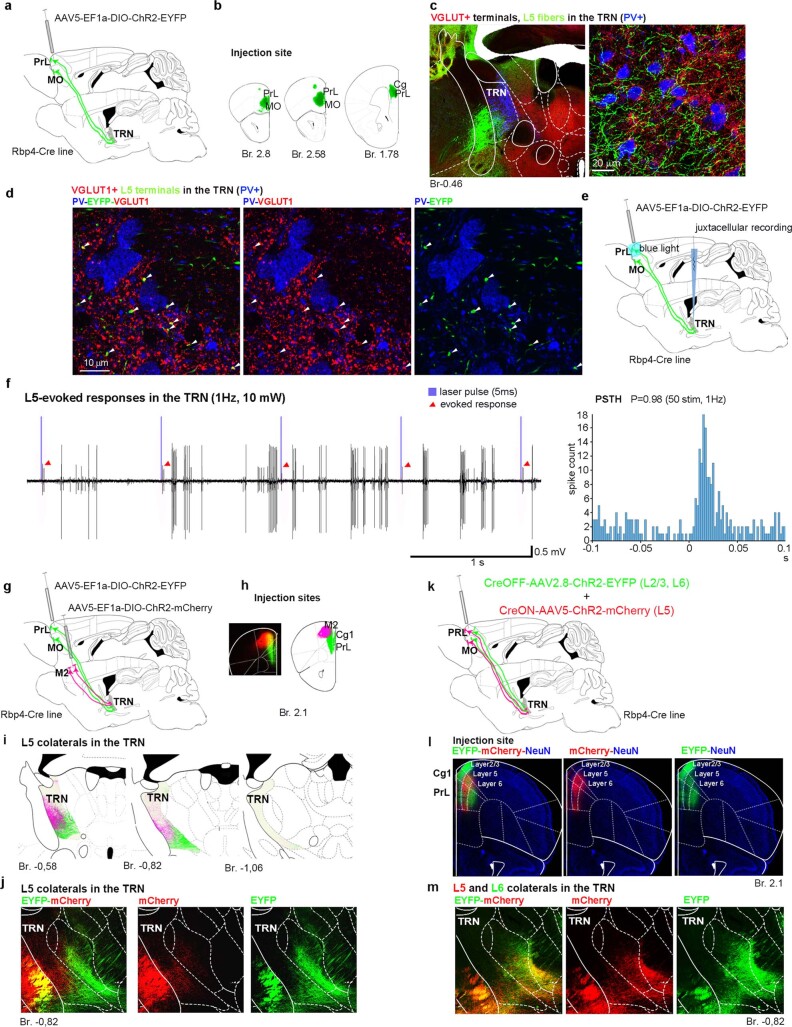
L5 innervation of the TRN from the mPFC. Related to Fig. 1. **a**) Experimental design. **b**) Schematic figure for injection site (mPFC). **c**) Confocal images of L5 collaterals (EYFP, green) in the TRN (PV + somas, blue). Vesicular Glutamate Transporter1 (VGLUT1), a marker for cortical synapses is labelled in red. **d**) High power confocal images of a single optical section (63x objective, z-step: 0.14 μm). Left panel: PV (blue), EYFP (green), VGLUT1 (red). Middle panel: PV (blue), VGLUT1 (red). Left panel: PV (blue), EYFP (green). Arrowheads point to VGLUT1 + L5 terminals. **e**) Experimental design. **f**) Left panel: Examples for juxtacellularly recorded TRN spike responses (red arrowheads) to optogenetic activation (duration of laser pulses marked with blue) of L5 cells in the mPFC. Right panel: Peristimulus time histogram (PSTH) (50 pulses at 1 HZ, response probability=0.98) of the same neuron. 0 marks the start of the laser pulses. **g**) Experimental design. **h**) Fluorescent image and schematic figure for injection sites (M2, mCherry, red; mPFC, EYFP, green). **i**) Schematic labelling of L5 fibers from the M2 (red) and the mPFC (green) in the TRN. **j**) Confocal images of the L5 fibers from the M2 (mCherry, red) and the mPFC (EYFP, green) in the TRN. **k**) Experimental design. (n = 2 mice). **l**) Injection site in the mPFC (mCherry, red, Cre-positive cells; EYFP, green, Cre-negative cells). Nuclear staining of the neurons (NeuN) is seen in blue. **m**) Confocal images of L5 (mCherry, red) and L6 (EYFP, green) collaterals in the TRN. Br: Bregma; Cg: cingulate cortex; M2: secondary motor cortex; MO: medial orbital cortex; Prl: prelimbic cortex; TRN: thalamic reticular nucleus.

To selectively label the axon arbor of the layer 5b (L5) pyramidal cells, floxed AAV5-EF1a-DIO-ChR2_EYFP virus was injected to the cortex of Rbp4–Cre mice (Fig. 1a–h). We locally labeled different cortical areas, specifically frontal associational areas (FrA), primary motor (M1) and secondary motor (M2), medial, lateral and ventral orbitofrontal (MO, LO and VO) cingulate (Cg) and prelimbic (Prl) cortices. In this study, we collectively refer to these regions as frontal cortex^26^. We also injected parietal (primer, S1 and secondary S2, somatosensory cortex), visual (including primer, V1 and secondary, V2 visual cortex) and insular cortical areas. Conditional viral tracing from the latter regions labeled only passing L5 fibers in the TRN, as described previously^23^ (Fig. 1a–c and Extended Data Fig. 1a–c). In contrast, frontal cortex areas provided dense L5 collaterals in the anterior part of the TRN studded with boutons (Fig. 1d–f and Extended Data Figs. 1d–i and 2a–d,g–m). To confirm the presence of the L5–TRN terminals in another mouse line, we mapped the distribution of EYFP^+^ terminals in the TRN of the Thy1-ChR2-EYFP mice (Fig. 1i–m), where EYFP is expressed in the L5 cells of the entire neocortex, including regions not targeted by our tracing experiments. Confirming the tracing data, we found labeled Thy1-ChR2-EYFP boutons only in the anterior but not in the posterior TRN sectors (Fig. 1j). The vast majority of the detected boutons were positive for vesicular glutamate transporter 1 (VGLUT1) and negative for vesicular glutamate transporter 2 (VGLUT2) (Fig. 1k–m), supporting their cortical origin.

To test whether L5 boutons in the anterior TRN form functional connections, we optogenetically activated M1/M2 (and, in one case, PrL) L5 cells from the cortical surface (5×10 pulses, 5 ms, 10 mW, at 1 Hz) in anesthetized (ketamine–xylazine) Rbp4–Cre mice injected with AAV5-EF1a-DIO-ChR2-EYFP virus (Fig. 1n–r and Extended Data Fig. 2e–f). We recorded the evoked responses of TRN cells using the juxtacellular recording and labeling method^27^. All TRN cells recovered post hoc were located in the anterior TRN surrounded by labeled L5 fibers (Fig. 1o). The TRN cells responded with short-latency and high-fidelity action potentials (Fig. 1p,r). These data suggest functional monosynaptic connection between the cortical L5 and the anterior TRN.

**Extended Data Fig. 3:**
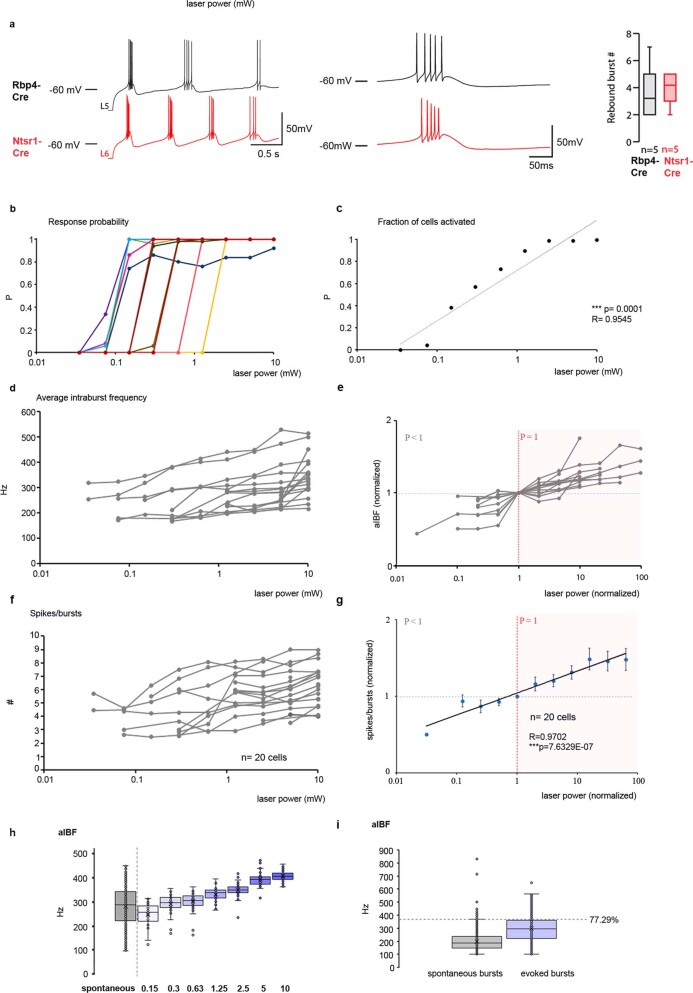
Properties of evoked cortical and TRN responses. Related to Fig. 1, Fig. 4 and Fig. 6. **a**) Left: *In vitro* current-clamp recordinging (Rbp4-Cre (top) and Ntsr1-Cre (bottom) mouse) showing TRN bursts upon release from hyperpolarized state. Middle: first burst, expanded view. Right: Number of rebound bursts, box plot (Rbp4-Cre: n = 5 neurons in n = 4mice; NTSR1-Cre: n = 5 neurons in n = 3mice). **b**) Response probabilities of L5 cells (n = 13 cells in n = 5 mice) at different laser powers. **c**) Population average of response probabilities for L5 cells (n = 13 cells in n = 5 mice). (Pearson correlation: R = 0.9545, *** p = 0.0001). **d**) Average intraburst frequency (aIBF) at different laser powers for TRN cells (n = 20 cells in n = 9 mice). **e**) aIBFs at different laser power values for TRN cells (n = 20 cells in n = 9 mice). Both aIBFs and power were normalized to the values observed at the minimal laser power at which response probability=1 (threshold value). **f**) Same as (d), spikes per burst. **g**) Spikes/burst values showed significant log-linear correlation with the laser power (n = 20 cells in n = 9 mice; Pearson correlation: R = 0.9702, ***p = 7.6329E-07). Error bars: average ± SEM. aIBFs and power values are normalized to the threshold value. . **h**) aIBFs of the spontaneous and evoked bursts at increasing laser power. Example cell, box plots (baseline: n = 649 bursts; 0.15 mW: n = 22 bursts; 0.3 mW: n = 37 bursts; 0.63 mW: n = 49 bursts; 1.25 mW: n = 50 bursts; 2.5 mW: n = 50 bursts; 5 mW: n = 50 bursts). au: arbitrary unit. Boxplots: box, first to third quartile; whisker’s ends, minimum and maximum values, x labels mean. Laser power is shown on logarithmic scale. **i**) Box plots for the aIBFs of spontaneous (n = 8666 bursts/17 cells) and evoked (n = 4236 bursts/17 cells) bursts (n = 9 mice). Dashed line: maximum value for the spontaneous bursts. aIBFs of 77.29 % of the optogenetically evoked bursts fall in the range of the values at baseline activity.

The recruitment of the anterior TRN cells by the L5 pathway was gradual. We tested the response probabilities of both L5 and TRN cells using different stimulation intensities in the cortex. The number of recruited L5 neurons increased with increasing laser power (Extended Data Fig. 3b,c). Although different L5 cells reached threshold at various laser intensities, they displayed all-or-none response probability curves (Extended Data Fig. 3b). By contrast, the response probabilities of the TRN cells increased gradually with increasing cortical stimulation power, and the response probability displayed significant correlation with the laser power on a logarithmic scale (Fig. 1r). These data together demonstrate the presence of a selective innervation of the anterior TRN from the L5 of the frontal cortex and imply the convergence of multiple L5 cells on a single TRN cell.

## L5–TRN collaterals are the side branch of corticofugal cells

**Fig. 2:**
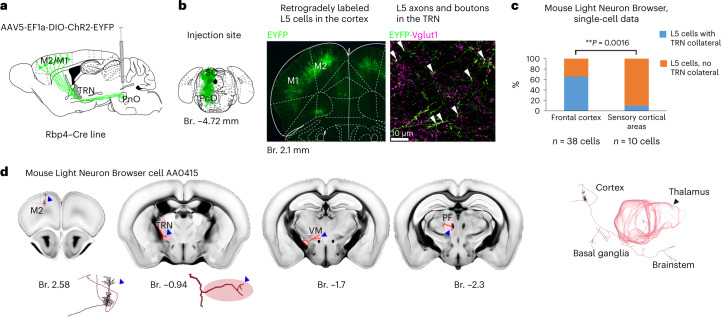
L5–TRN collaterals are the collaterals of frontal corticofugal cells. **a**, Experimental design (*n* = 3 mice). **b**, Left panel: injection site. Middle panel: fluorescent image of retrogradely labeled L5 cells in the frontal cortex. Right panel: confocal image of Vglut1^+^ cortical L5 terminals in the anterior TRN. **c**, Proportion of corticothalamic L5 cells in the frontal versus sensory cortices with and without TRN collaterals based on reconstructed L5 cells from the Mouse Light Neuron Browser database (*n* = 25/38 cells from the frontal cortex and *n* = 1/10 cells from the sensory–somatosensory, visual and auditory cortices; chi-square test: **P = 0.0016). **d**, An example L5 cell from the Mouse Light Neuron Browser. Left panel: position of the soma in the frontal cortex (islet: larger magnification of the cell body). Middle panels: collaterals in the anterior TRN (islet: larger magnification of the L5 collateral) and in the VM and Pf. Right panel: the complete reconstructed cell with its subcortical targets. Br., bregma; SC, superior colliculus.

To test if frontal L5 cells innervating the anterior TRN belong to a specific pyramidal cell population or have additional subcortical targets (like classical PT neurons), we used the retro-anterograde tracing method^28^. We injected AAV-EF1a-DIO-ChR2-EYFP virus to the upper brainstem of Rbp4–Cre mice (Fig. 2a,b) and allowed the mice to survive for 2–3 months. Using this method, the virus first spread retrogradely selectively in the brainstem-projecting Rbp4–Cre^+^ L5 cells, and then its product was transferred to their axonal collaterals in an anterograde manner (Fig. 2b). In all three mice, brainstem-projecting L5 cells displayed EYFP^+^ axon terminals in the anterior TRN. These terminals were positive for VGLUT1, confirming their cortical origin (Fig. 2b).

To test the presence of L5–TRN collaterals at single-cell level, we analyzed the cell reconstruction data of the Mouse Light Neuron Browser database^29^ (Fig. 2c,d and Supplementary Table 1) and found that 65.79% of thalamus-projecting L5 cells from the frontal cortex (M1/M2, Cg1 and orbitofrontal cortex) emitted collaterals to the anterior TRN (Fig. 2c,d), indicating a high frequency of L5–TRN collaterals in this population. In the case of L5 cells from the sensory cortices, only 10% had TRN collateral (Fig. 2c). All the TRN-projecting cells sent axons to the basal ganglia and the brainstem as well (Fig. 2d). These two datasets together confirm that TRN-projecting L5 cells belong to the classical brainstem-projecting PT cells.

## Parallel but morphologically distinct L5–TRN and L6–TRN pathways

**Fig. 3:**
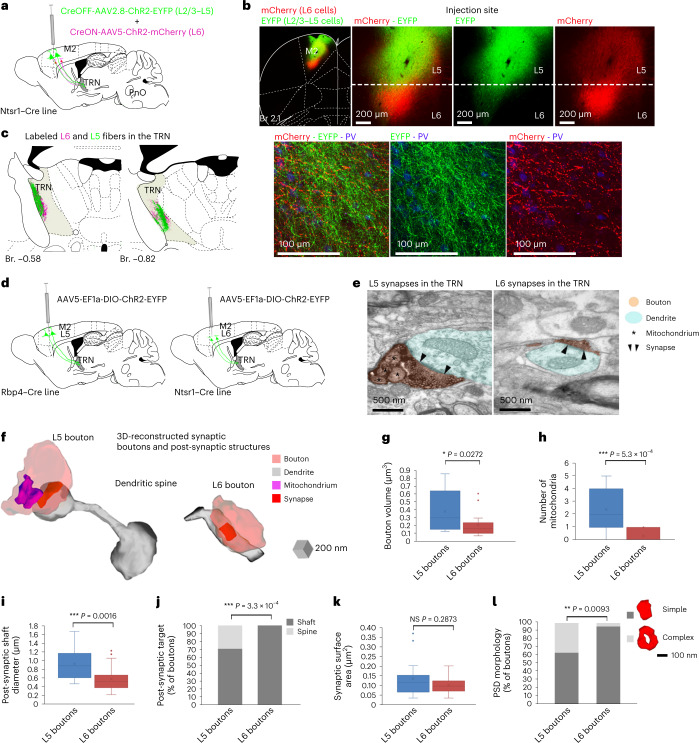
Parallel but distinct L5–TRN versus L6–TRN pathways. **a**, Experimental design (*n* = 3 mice). **b**, Low-power (left) and high-power (right panels) confocal images of the injection site (mCherry, red, Cre^+^ cells; EYFP, green, Cre^−^ cells). **c**, Left panels: L6 (magenta) and L5 (green) projection zones in the TRN. Right panels: confocal image of the L6 and L5 projection zones in the TRN (PV^+^, blue). **d**, Experimental design. **e**, Electron micrographs of an L5 (left panel) and an L6 (right panel) terminal (black precipitate, orange shading) establishing synapses on TRN dendritic shafts (blue shading). Arrowheads: PSDs; asterisks: mitochondria. **f**, Examples for 3D-reconstructed L5 (left) and L6 (right) boutons and their post-synaptic targets (L5: dendritic spine; L6: dendritic shaft) in the TRN. **g**, Box plots for the volumes of L5 and L6 boutons (L5: *n* = 14 boutons in *n* = 2 mice, 0.38 ± 0.07 µm^3^; L6: *n* = 13 boutons in *n* = 2 mice 0.2 ± 0.05 µm^3^; Mann–Whitney *U*-test: *P = 0.0272). **h**, Box plots for number of mitochondria in the L5 and L6 boutons (L5: *n* = 15 in *n* = 2 mice, 2.4 ± 0.46; L6: *n* = 15 in *n* = 2 mice, 0.33 ± 0.13, Mann–Whitney *U*-test: ***P = 5.3 × 10^−4^). **i**, Box plots for the diameter of post-synaptic dendritic shafts for L5 versus L6 terminals in the TRN (L5: *n* = 12 in *n* = 2 mice, 0.94 ± 0.1 µm; L6: *n* = 40 in *n* = 2 mice; 0.59 ± 0.04 µm, Mann–Whitney *U*-test: **P = 0.0016). **j**, Post-synaptic targets of L5 (*n* = 17 in *n* = 2 mice) versus L6 (*n* = 40 in *n* = 2 mice) terminals in the TRN (L5 boutons targeting dendritic spines: 29.41%; L6 boutons targeting dendritic spines: 0%, chi-square test: ***P = 3.293 × 10^−4^). **k**, Box plots for the synaptic surface area of the L5 and L6 synapses in the TRN (L5: *n* = 19 in *n* = 2 mice 0.14 ± 0.02 µm^2^; L6: *n* = 24 in *n* = 2 mice 0.1 ± 0.01 µm^2^, Mann–Whitney *U*-test: NS *P* = 0.2873). **l**, Morphological types of the PSD in the L5 (*n* = 19 in *n* = 2 mice) and L6 (*n* = 22 in *n* = 2 mice) synapse populations in the TRN (L5 boutons forming complex synapse: 36.84%; L6 boutons forming complex synapse: 4.55%, chi-square test: **P = 0.0093). Box plots: box shows the first to third quartiles; whisker ends indicate minimum and maximum values; x labels the mean. Br., bregma.

Frontal cortex areas are known to target the TRN via L6 pyramidal cells^30^. Thus, we examined whether L6 and L5 inputs from the same cortical area form parallel or divergent pathways in the TRN and whether they are morphologically and/or functionally different. To simultaneously label both pathways, we injected a mixture of CreON and CreOFF viruses to the M2 of the L6-specific Ntsr1–Cre mouse (Fig. 3a). At the injection site, the AAV-EF1a-DIO-ChR2-mCherry virus (CreON, red) was expressed selectively in the L6 corticothalamic cells in a Cre-dependent manner, whereas the AAV-DFO-ChR2-eYFP virus (CreOFF, green) was expressed exclusively in the cortical cells that did not contain the Cre-recombinase (including the L5 corticothalamic cells). Because thalamus receives cortical inputs only from L6 and L5 corticothalamic cells^31^, we could reliably label the two populations in parallel within the same animal using this method (Fig. 3b). In all three animals, L6 and L5 projection zones displayed strong overlap in the anterior TRN (Fig. 3c), implying that L6 and L5 inputs from the same cortical source converge in the same TRN zone. In Rpb4–Cre mice, we injected the CreON–CreOFF virus mix to the PrL/Cg. Again, L6 and L5 projection zones strongly overlapped in the TRN (Extended Data Fig. 2k–m).

The functional properties of a synapse are closely related to the ultrastructure of its pre-synaptic and post-synaptic elements. Thus, we compared L6 and L5 synapses at electron microscopic (EM) level. L6 and L5 cells were virally labeled in the M1/M2 cortex of the Ntsr1–Cre and Rbp4–Cre mouse lines, respectively (Fig. 3d). Both L5 and L6 terminals established classical asymmetrical synapses in the TRN (Fig. 3e). We analyzed serial EM sections of the labeled L6 and L5 synapses and reconstructed the boutons and their post-synaptic partners in three dimensions (Fig. 3f). L5 boutons had significantly larger volume (Fig. 3g) and contained, in most cases, multiple mitochondria. By contrast, L6 boutons contained no or a maximum of one mitochondrion (Fig. 3h). L5 boutons targeted significantly thicker dendrites compared to the L6 boutons, suggesting that they may prefer different dendritic compartments (Fig. 3i). In line with this, 29.41% of the L5 boutons targeted dendritic spines, whereas L6 boutons targeted exclusively dendritic shafts (Fig. 3j). Although there was no significant difference between the synaptic surface area of the L5 and L6 synapses (Fig. 3k), 36.84% of the L5 synapses had complex (perforated or branching) morphology, whereas L6 synapses showed, in all but one case (4.55%), simple, discoid morphology (Fig. 3l). These data show that the L6–TRN and L5–TRN pathways have different ultrastructural characteristics regarding both the pre-synaptic and post-synaptic elements, suggesting that they have distinct functional properties.

## Distinct physiological properties of the L5–TRN and L6–TRN pathways

**Fig. 4:**
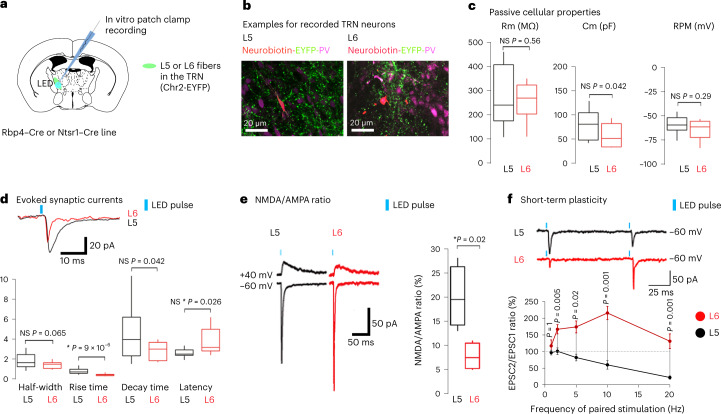
Distinct physiological properties of the L5–TRN and the L6–TRN pathways. **a**, Experimental design. **b**, Filled TRN cells in the TRN (PV^+^) among L5 (left) or L6 (right) fibers (EYFP). **c**, Box-and-whisker plots showing the membrane resistance (Rm), membrane capacitance (Cm) and resting membrane potential (RMP) of TRN neuorns in Rbp4–Cre (L5, black) and Ntsr1–Cre (L6, red) mice (L5: *n* = 18 cells in *n* = 10 mice, L6: *n* = 10 cells in *n* = 4 mice; Rm: 287 ± 30 MΩ versus 264 ± 25 MΩ, Cm: 81 ± 8 pF versus 58 ± 8 pF, RMP: −59 ± 2 mV versus −64 ± 4 mV; unpaired Student’s *t*-tests, *P* = 0.56 for Rm, *P = 0.042 for Cm and *P* = 0.29 for RMP). **d**, Box plots showing the evoked EPSCsʼ half-width, rise time, decay time and latency from LED onset for L5 (black) and L6 (red) synapses (L5: *n* = 15 cells in *n* = 8 mice, L6: *n* = 9 cells in *n* = 4 mice; half-width: 1.88 ± 0.21 ms versus 1.41 ± 0.12 ms; rise time: 0.8 ± 0.09 ms versus 0.43 ± 0.04 ms; decay time: 4.67 ± 0.76 versus 2.87 ± 0.30 ms; latency: 2.62 ± 0.12 versus 3.86 ± 0.45 ms; unpaired Student’s *t*-test, P=0.065 for half-width, ***P = 9 × 10^−4^ for rise time, *P = 0.042 for decay time, *P = 0.026 for latency). Islet: examples for L5 and L6 EPSCs. **e**, Left: typical traces of NMDA/AMPA-mediated synaptic responses. Bottom traces: evoked EPSCs at −60 mV in artificial cerebrospinal fluid (ACSF). Top traces: NMDAR-mediated currents at +40 mV in ACSF and DNQX. Right: box plots of the NMDA/AMPA ratio in L5 and L6 synapses (L5: *n* = 4 cells in *n* = 4 mice and L6: *n* = 4 cells in *n* = 1 mouse; 20 ± 3% versus 8 ± 1%; unpaired Student’s *t*-test, *P = 0.02). **f**, Top: typical traces from voltage-clamp recording of TRN neurons upon paired light activation of L5 (black) and L6 (red) afferents. Bottom: paired-pulse ratio of EPSCs in TRN neurons at 1, 2, 5, 10 and 20 Hz (L5: *n* = 7 cells in *n* =  6 mice, L6: *n* = 10 in *n* = 4 mice; Wilcoxon rank-sum test with Bonferroni correction for multiple comparison (significance at 0.01)). Error bars depicts average ± s.e.m. Box plots: box shows first to third quartiles; whisker ends indicate minimum and maximum values; x labels the mean.

To compare the synaptic properties of L5–TRN and L6–TRN pathways, we used in vitro electrophysiology experiments. AAV5-EF1a-DIO-ChR2-EYFP virus was injected 2–4 weeks before the experiments in the M2 cortex of Ntsr1–Cre or Rbp4–Cre mice to label L6 and L5 pathways, respectively. L5 and L6 fibers were optogenetically activated while TRN cells were recorded in whole-cell patch clamp configuration (Fig. 4a,b). TRN neurons in Rbp4–Cre and Ntsr1–Cre mice showed similar passive cellular properties (Fig. 4c). TRN cells of both mouse strains showed multiple rebound burst discharge upon release from a hyperpolarized state (Extended Data Fig. 3a). Optogenetic activation of L5 afferents induced excitatory post-synaptic currents (EPSCs) that were, on average, three times larger than those evoked by stimulation of L6 afferents (L5: *n* = 18 neurons in *n* = 10 mice; L6: *n* = 10 neurons in *n* = 4 mice; −74 ± 13 pA versus −25 ± 8 pA; Wilcoxon rank-sum test, **P = 0.003). The rise and decay times of the L5–TRN EPSCs were significantly longer compared to the L6–TRN EPSCs, and the response latency was shorter in the case of the L5–TRN pathway (Fig. 4d). The L5–TRN EPSCs had significantly higher NMDA-receptor-mediated component (Fig. 4e).

The short-term plasticity of the L5–TRN and L6–TRN pathways displayed opposite features. Although paired-pulse activation of L5 afferents showed clear short-term depression, L6 activation showed typical short-term facilitation described previously for L6 projections from the somatosensory cortex^32^ (Fig. 4e).

In summary, L5–TRN synapses had higher NMDAR content than L6–TRN synapses, which might explain differences in the EPSC time course, notably its decay time. Moreover, the two pathways showed different forms of short-term plasticity. Strong short-term depression of the L5–TRN pathway implies that it may be better tuned for the integration of instantaneous and synchronous L5 activity than to faithfully follow long spike trains.

## Segregation and integration of L5 inputs in the TRN

**Fig. 5:**
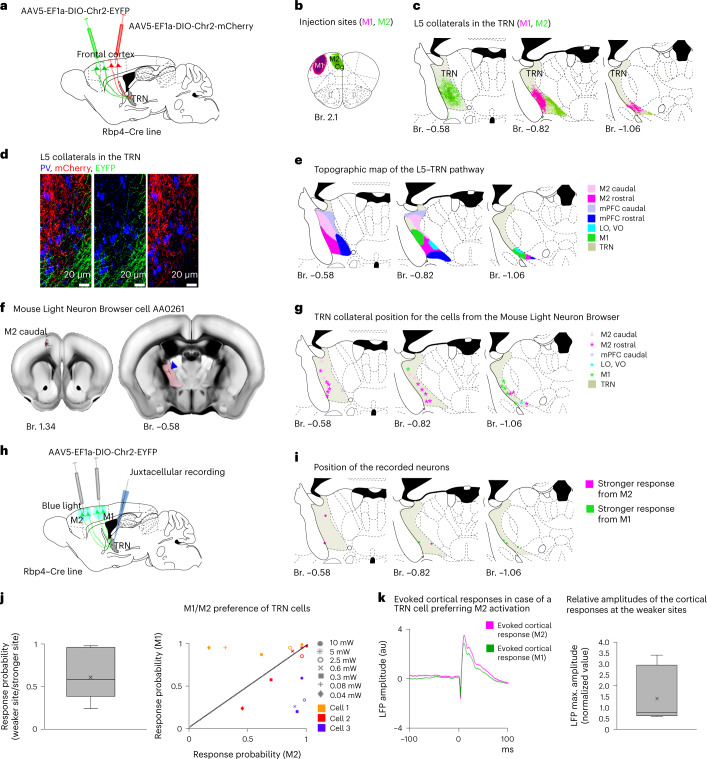
Topography of the L5–TRN pathway. **a**–**c**, Experimental design (**a**), injection sites (**b**) and projection zones in the TRN (**c**). **d**, Confocal images of the non-overlapping M1 and M2/Cg L5 collaterals in the TRN (PV^+^). **e**, Topographical map of the frontal L5 to anterior TRN projection (*n* = 13 injections: M2 rostral + M2 caudal (*n* = 1); M2 rostral + M1 (*n* = 1); M2 rostral + mPFC rostal (*n* = 1); mPFC rostral (*n* = 3); mPFC caudal (*n* = 2); orbitofrontal cortex (*n* = 2); M1 (*n* = 3)). **f**, A reconstructed L5 corticothalamic cell from the Mouse Light Neuron Browser. Left: position of the cell body in the caudal M2. Right panel: position of axon collateral (red) in the TRN (labeled with pale red). **g,** Axon collateral positions (*n* = 26) of *n* = 22 reconstructed L5 corticothalamic cells in the TRN. Colors (right) indicate the position of the soma. Note the close correspondence between the viral tracing (**e**) and the single-cell labeling data. **h,** Experimental design. **i**, Positions of the recorded TRN cells. Colors indicate the site of cortical activation with stronger TRN responses. **j,** Left: box plot depicting the ratio of TRN response probabilities from cortical stimulation sites with the lower versus higher TRN responses normalized to the response probabilities from the stronger site (*n* = 7 cells in *n* = 5 mice, P weaker/stronger site: 0.61 ± 0.1). Right: comparison of the response probabilities after M1 versus M2 stimulation for a sample of three TRN cells at different laser powers. Symbols indicate power values; colors label different cells. **k**, Left: an example (cell 3 from **j**) for evoked cortical response averages at the sites with the weaker (M1) and with the stronger (M2) TRN responses. Right: box plot for peak amplitudes of the evoked cortical response averages at the sites with the weaker TRN response probabilities normalized to the response peak amplitudes at the stronger cortical sites (*n* = 7 cells in *n* = 5 mice, 1.40 ± 0.46 au). Box plots: box shows first to third quartiles; whisker ends indicate minimum and maximum values, x labels the mean. au, arbitrary units; Br., bregma; LO, lateral orbital cortex; VO, ventral orbital cortex.

The previous in vivo electrophysiology experiments (Fig. 1n–r and Extended Data Fig. 3b) implied the convergence of multiple L5 cells on a single TRN cell. Therefore, we asked to what extent the frontal cortex L5 activity could be integrated at the level of single TRN cells. To address this question, double viral injections (AAV5-EF1a-DIO-ChR2-EYFP and AAV5-EF1a-DIO-ChR2-mCherry) with non-overlapping injection sites were made to various combinations of frontal cortex regions in Rbp4–Cre mice (Fig. 5a,b and Extended Data Fig. 2g,h). In all cases, we saw clear segregation of L5 collaterals arising from neighboring cortical regions in the TRN (Fig. 5c,d and Extended Data Fig. 2i,j). Using the results of multiple double and single viral labeling experiments, we created the map of the L5–TRN pathway (Fig. 5e). We found segregated, patchy organization of L5–TRN termination zones. As an indication for a topographical organization, we found that, within one cortical region, axons from more caudal cortical areas targeted preferentially more dorsal parts of the TRN compared to the axons from the more rostral areas. We compared these tracing data with single-cell results of the Mouse Light Neuron Browser database. Areal localization of the corticothalamic cells and the position of their TRN collaterals were consistent with our viral tracing data (Fig. 5f,g).

Next, we tested whether single TRN neurons are able to integrate inputs from two different cortical regions in the intact brain by stimulating L5 cells in the M1 and M2 areas (same rostrocaudal but different mediolateral level) in Rbp4–Cre mice (Fig. 5h,i). All juxtacellularly recorded TRN neurons showed evoked responses (single spikes or bursts depending on the stimulation intensity) from both cortical regions. The preferred stimulation site for each cell was determined as the site with higher response probability (P) at 10-mW stimulus power (Fig. 5j). We found cells with strong M1 preference, cells with strong M2 preference and cells with similar response probabilities for the two stimulation sites (Fig. 5j). Matching our viral tracing data (Fig. 5e), somata of TRN cells with stronger response to M1 stimulation were positioned in more-caudal TRN regions compared to TRN cells that preferred M2 stimulation (Fig. 5i). Evoked cortical local field potential (LFP) responses were of similar magnitude at the sites evoking strong or weak responses in TRN, indicating that the preference of the TRN cells was not due to uneven activation of cortical sites (Fig. 5k).

These data together demonstrate that L5 inputs from different cortical regions segregate at the level of the TRN. However, individual TRN cells are able to integrate inputs from different cortical regions, probably through their extensive dendritic arbors spanning multiple cortical termination zones.

## TRN spike output reflects gradual recruitment of L5 inputs

Relatively weak L5-mediated synaptic responses in vitro (Fig. 4d,f) could elicit reliable responses in the TRN in vivo (Fig. 1q–r), often from multiple cortical origins (Fig. 5h–k). This suggests a significant amount of convergence in the L5–TRN pathway. This was further supported by the gradual recruitment of TRN cells with increasing laser intensities (Fig. 1r) in contrast to the all-or-none responses of L5 cells (Extended Data Fig. 3b,c). This implies that convergent, individually weak L5 inputs might enable TRN cells to faithfully read out gradual changes in cortical population activity.

**Fig. 6:**
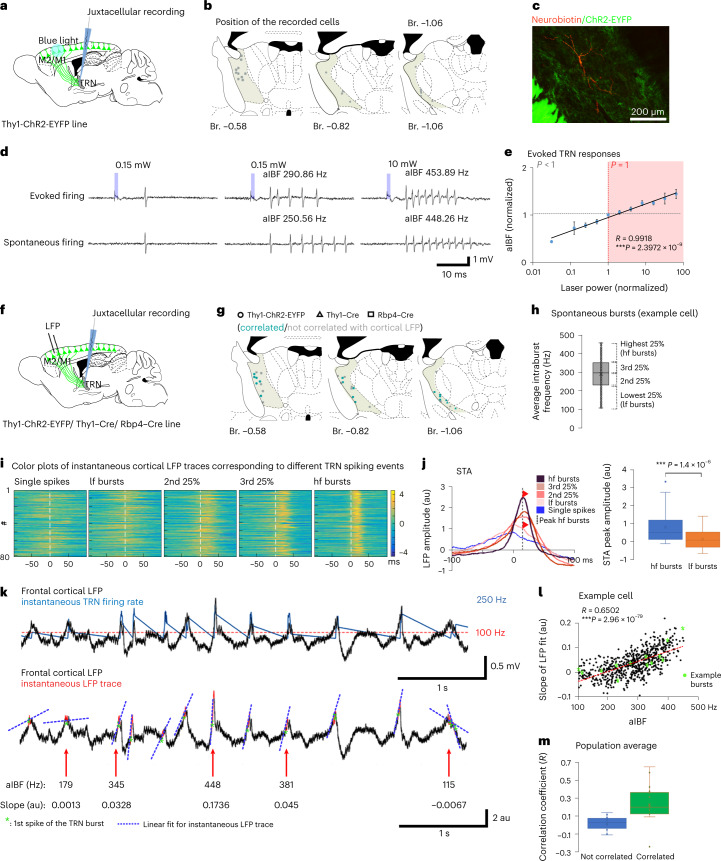
Instantaneous TRN firing–cortical LFP correlation. **a**, Experimental design. **b**, Recorded cells. **c**. Neurobiotin-filled TRN cell, L5 fibers (Chr2-EYFP). **d**, Evoked and spontaneous firing of the same TRN cell. Blue: laser stimulus (power indicated). **e**, Evoked aIBFs for *n* = 20 cells in *n* = 9 mice at different laser powers. aIBFs and power values are normalized to the value observed at the minimal power at which the response probability = 1. Correlation between aIBF and stimulation power (Pearson correlation: *R* = 0.9918, ***P = 2.3972 × 10^−9^). Error bars depicts average ± s.e.m. **f**, Experimental design. **g**, Recorded cells. Circle: Thy1-ChR2-EYFP; triangles: Thy1-Cre; squares: Rbp4–Cre line. **h**, Grouping of spontaneous bursts along the aIBF quartiles (lf, low-frequency; hf, high-frequncy). **i**, Color plots of instantaneous LFP traces for single spikes and different classes of bursts (as shown in **h**) of an example TRN cell. Colors indicate amplitude. Rows represent single firing events. 0 ms: single spike or first spike of burst. Equal numbers of events for all categories were selected randomly. **j**, Left: STAs (same cell as in **i**). Dashed line: STA peak for hf bursts. Red arrowheads: STA amplitudes for lf and hf bursts. Right: box plots (*n* = 31 cells in *n* = 13 mice) for STA peaks of lf and hf bursts (peak amplitudes: 0.14 ± 0.11 versus 0.81 ± 0.14 au; Student’s paired *t*-test: ***P = 1.4 × 10^−6^). **k**, Upper panel: cortical LFP trace (black) and instantaneous firing rate (blue) of a TRN cell. Red dashed line: burst threshold. Lower panel: linear fit for the instantaneous LFP trace (30-ms-long LFP sections around the first spikes). Green asterisk: first spikes of the bursts. Red: instantaneous LFP trace. Blue dashed lines: linear fits. aIBFs and the LFP slope values are indicated for each burst. **l**, aIBF–LFP slope correlation for *n* = 649 bursts of the example neuron (Pearson correlation: *R* = 0.6502, ***P = 2.96 × 10^−79^). Green asterisks: example bursts on **k**. **m**, Box plots for the correlation coefficients (*R*) for aIBF–LFP slope correlations (*n* = 31 cells in *n* = 13 mice). Green: significant correlation (*n* = 19 cells; *R* = 0.230 ± 0.051); blue: no correlation (*n* = 12 cells; *R* = 0.027 ± 0.022). Box plots: box shows first to third quartiles; whisker ends indicate minimum and maximum values, x labels the mean. au, arbitrary units; Br., bregma.

To test this idea, we optogenetically increased cortical (M1/M2) L5 population activity step by step and examined the properties of the evoked TRN responses in the Thy1-ChR2-EYFP mice (Fig. 6a–e). Evoked responses ranged from single spikes to bursts with up to 564-Hz average intraburst frequencies (aIBFs) (Fig. 6d and Extended Data Fig. 3d,e). Individual bursts contained 2–16 spikes (Extended Data Fig. 3f,g). Single spike events and bursts with broad range of aIBFs (100–369 Hz) (Fig. 6d and Extended Data Fig. 3h,i) could also be observed during the spontaneous activity of the same cells. aIBFs of 77.29% of the evoked bursts were in the range of the values observed during baseline activity (Extended Data Fig. 3h). This indicates that most of the optogenetically evoked activity was within the physiological range.

Both the aIBFs and the number of spikes per evoked bursts showed significant log-linear correlation with the cortical L5 stimulus power (Fig. 6e and Extended Data Fig. 3d–g). Because the fraction of recruited L5 cells showed similar, log-linear correlation with the laser power (Extended Data Fig. 3b,c), these data demonstrate that the exact spike output of the TRN cells reflects the number of simultaneously recruited L5 cells.

## Instantaneous correlation between cortical and TRN activity

**Extended Data Fig. 4:**
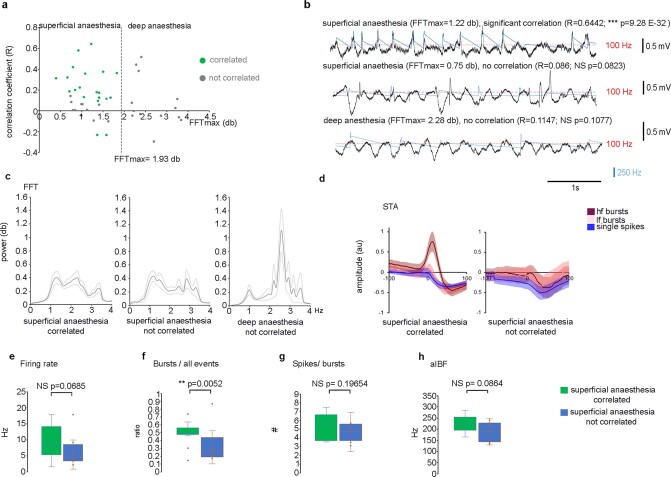
Correlation between the spontaneous burst properties and the instantaneous cortical LFP at different depths of anaesthesia. Related to Fig. 6. **a**) Relationship of the aIBF-LFP slope correlation coefficient and the peak amplitude of the fast Fourier transform (FFTmax) of the frontal LFP at 1–4 Hz (n = 44 cells). Green dots: TRN cells with significant correlation. Grey dots: cells with no correlation. Dashed line: superficial–deep anaesthesia boundary (at average ± 2 SD of the FFTmaxs using recordings with significant aIBF-LFP slope correlation). **b**) Cortical LFP traces, and instantaneous firing rates of sample TRN neurons for superficial anaesthesia with significant aIBF-LFP correlation (upper panel) (FFTmax = 1.22 db; Pearson’s Correlation: R = 0.6442; ***p = 9.28E-32, n = 649 bursts), for superficial anaesthesia with no significant aIBF- LFP correlation (middle panel) (FFTmax = 0.75 db; Pearson’s Correlation: R = 0.086; NS p = 0.0823; n = 409 bursts) and for deep anaesthesia with no significant aIBF- LFP correlation (lower panel) (FFTmax = 2.28 db; Pearson’s Correlation: R = 0.1147; NS p = 0.1077; n = 198 bursts). Dotted line: burst threshold. **c**) FFT spectrum (average ± SEM) for recordings with superficial anaesthesia and significant aIBF-LFP correlation (n = 19 cells in n = 11 mice, left), for recordings with superficial anaesthesia and no significant aIBF- LFP correlation (n = 12 cells in n = 7 mice, middle) and for recordings with deep anaesthesia and no significant aIBF-LFP correlation (n = 13 cells in n = 8 mice, right). Note the peak in the delta frequency range on the right panel. **d**) Spike triggered LFP averages (STAs) ±SEM for single spikes, lf bursts, and hf burst. Left: superficial anaesthesia, significant aIBF-LFP slope correlation (n = 19 cells in n = 11 mice). Right: superficial anaesthesia, no significant aIBF- LFP correlation (n = 12 cells in n = 7 mice). **e**) Firing rate for cells with superficial anaesthesia and significant aIBF-LFP correlation (9.17 ± 1.17 Hz, n = 19 cells in n=11 mice), superficial anaesthesia, no significant aIBF- LFP correlation (6.06 ± 1.32 Hz, n = 12 cells in n=7 mice) (Mann-Whitney U Test: NS p = 0.0685). **f**) Same as (e) for burst rate. Superficial anaesthesia, significant aIBF-LFP correlation (n = 19 cells in n=11 mice): 0.5 ± 0.03; superficial anaesthesia, no significant aIBF-LFP correlation (n = 12 cells in n=7 mice): 0.34 ± 0.05; Mann-Whitney U Test: ** p = 0.0052). **g**) Same as (e) for spikes/bursts. Superficial anaesthesia, significant aIBF-LFP correlation (n = 19 cells in n=11 mice; 5.36 ± 0.34); superficial anaesthesia, non-significant aIBF-LFP correlation (n = 12 cells in n=7 mice, 4.67 ± 0.36; Mann-Whitney U Test: NS p = 0.2). **h**) Same as (e) for aIBF. Superficial anaesthesia, significant aIBF-LFP correlation (n = 19 cells in 11=mice): 223.72 ± 8.90; superficial anaesthesia, no significant aIBF-LFP correlation (n = 12 cells in n = 7 mice): 188.85 ± 12.93; Mann-Whitney U Test: NS p = 0.0864. au: arbitrary unit; boxplots: box, first to third quartile; whisker’s ends, minimum and maximum values, x labels mean.

To investigate whether TRN spiking was also modulated by spontaneous changes in the cortical synchrony, we recorded the spontaneous (baseline) activity of TRN cells in parallel with the frontal (M2) cortical LFP (Fig. 6f). Synchronous cortical population activity similar to the optogenetically evoked cortical responses could be detected in the LFP recordings as transient, fast, high-amplitude events. Under our conditions, fast LFP transients were present only in light, but not in deep, anesthesia (Extended Data Fig. 4). Thus, we used the recordings under light anesthesia for further analysis (31 of 44 cells) (Methods and Fig. 6g).

Examination of individual TRN spike-triggered LFPs and spike-triggered averages (STAs) of LFPs showed that spontaneous single spikes fired by TRN cells were mostly associated with irregular cortical activity. In contrast, TRN bursts were associated with fast LFP transients (Fig. 6i–j and Extended Data Fig. 4d). Bursts with higher aIBFs had a population STA with progressively higher peak amplitude, indicating that faster bursts are better synchronized with higher-amplitude cortical events compared to slower bursts (Fig. 6i–j).

To quantify the gradual, instantaneous relationship between cortical population activity and TRN firing, we correlated the aIBFs of the individual TRN bursts and the slope of the corresponding cortical LFP transients (Methods and Fig. 6k–m). In 19 of 31 TRN cells (61.3%), there was a significant correlation between the aIBF of the bursts and the magnitude of the instantaneous LFP slope. In 12 of the 31 TRN neurons (38.7%), either bursts were not associated with cortical LFP transients (Extended Data Fig. 4b,d) or the burst properties were not correlated with the magnitude of the slope of the cortical events (Fig. 6m). TRN cells with or without significant aIBF–LFP slope correlation were mixed spatially within the anterior TRN (Fig. 6g), which indicates heterogeneous association of cortical and TRN activity within a TRN sector. Spontaneous firing properties of the two populations were not significantly different, except that burst rate was higher for cells with significant aIBF–LFP slope correlation (Extended Data Fig. 4e–h).

TRN bursts with higher aIBFs were correlated with faster LFP events. These data clearly demonstrate a tight link between cortical population activity and the exact spiking output of most TRN cells.

**Extended Data Fig. 5:**
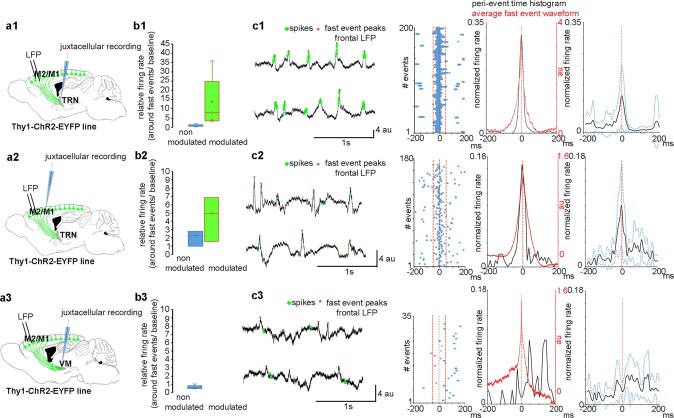
Modulation of anterior TRN cell, frontal cortical L5b pyramidal and VM cell firing by fast cortical transients. a1–3) Schematics of experimental design to record frontal cortical LFP and TRN (a1), L5 (a2) and VM (a3) unit activity. (b1) Firing rate of TRN cells around the fast cortical event peaks (±50 ms) relative to the baseline activity (50–200 ms before and after the peak). (n = 12 cells in n = 5 mice) Blue: cells whose firing was not modulated around the peak (n = 6 cells) (relative firing rate: 0.99 ± 0.24). Green: cells whose firing was positively modulated around the peak (n = 6 cells) (relative firing rate: 11.48 ± 5.16). (b2) Same as (b1) for L5b cells (n = 6 cells in n = 2 mice). Relative firing rate for not modulated cells (n = 3): 2.04 ± 0.55; relative firing rate for modulated cells (n = 3): 4.48 ± 1.55. (b3) Same as (b2) for VM cells (VM, n = 5 cells in n = 5 mice). Relative firing rate for not modulated cells (n = 5): 3.51 ± 0.1. (c1) Left panel, Spiking activity of an example TRN cell in the ±200 ms time window around the peak of the detected fast cortical events; middle panel, peri-event time histogram for an example cell (black). Average fast event waveform (red); right panel, population peri-event time histogram (n = 13 cells). Average (black) and ±std (blue dotted lines). (c2) Same as (c1) for L5b cells (n = 6 cells). (c3) Same as (c1) for VM cells (n = 5 cells). Boxplots: box, first to third quartile; whisker’s ends, minimum and maximum values, x labels mean. au: arbitrary unit.

Synchrony of TRN and cortical activity can arise not only from L5 neurons as proposed here but also from the relay cells, which innervate both the TRN and the cortex. To address this question, we recorded the activity of TRN cells; thalamocortical cells in the ventromedial relay nucleus, which has frontal cortex connections; and L5 cortical neurons in the frontal cortex in Thy1-ChR2-EYFP mice. We compared the activity of these cells during the fast cortical LFP transients (Methods). Here, six of the 12 TRN cells elevated their firing around the peaks (± 50 ms) of the transients (Extended Data Fig. 5a1–c1). Three of six L5 cells (*n* = 2/3 cells in M2, *n* = 1/3 cells in PrL) also strongly modulated their activity during the fast transients (Extended Data Fig. 5a2–c2). However, we did not see elevated spiking activity at the fast LFP transients in the VM population (*n*=5 cells) (Extended Data Fig. 5a3–c3). These data suggest that the elevated cortical, not thalamic, firing underlies the recruitment of TRN bursting at fast cortical transients.

Taken together, we found that, in the case of both the evoked and spontaneous cortical population events, the exact spike output of the anterior TRN cells provides a gradual readout of the magnitude of synchronous cortical activity.

## The L5–TRN pathway conveys cortical synchrony to TRN

**Fig. 7:**
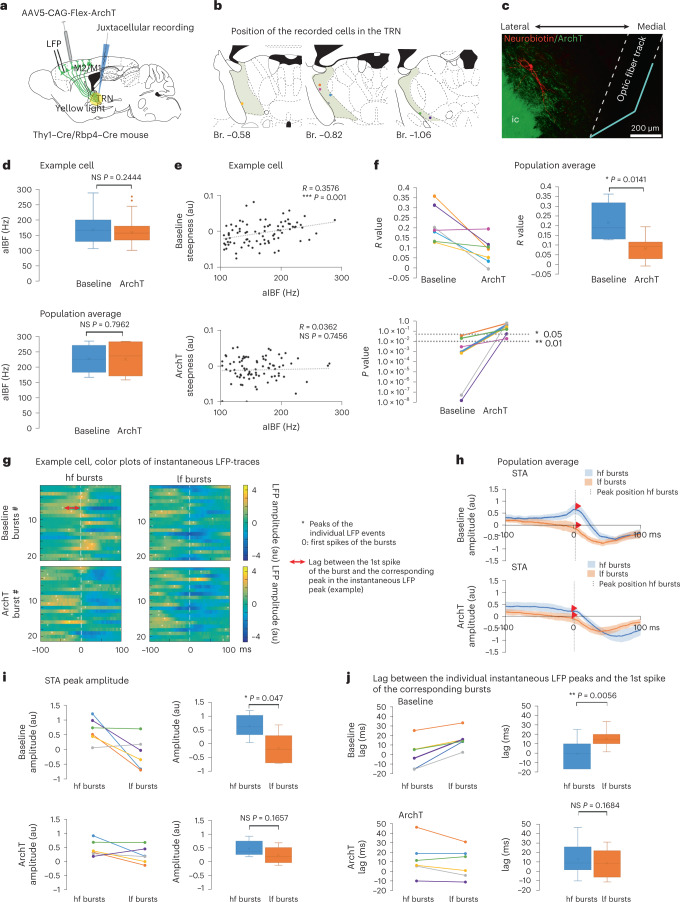
L5–TRN pathway conveys cortico–TRN correlation. **a**, Experimental design. **b**, Recorded cells. Colors as in **f**,**i**,**j**. **c**, Neurobiotin-filled cell, L5 fibers (ArchT-EYFP). Dashed line: optic fiber. Blue line: mirror. **d**, Box plots for aIBFs during baseline and ArchT conditions. Upper panel: example cell (166 ± 4.7 Hz versus 159.08 ± 1.87 Hz; Mann–Whitney *U*-test: *P* = 0.2444) (baseline: *n* = 83 bursts; ArchT: *n* = 313 bursts). Lower panel: *n* = 7 cells in *n* = 4 mice (*n* = 2 Rbp4–Cre, *n* = 2 Thy1–Cre) (227.25 ± 16.63 Hz versus 225.49 ± 19.59 Hz; Student’s paired *t*-test: *P* = 0.7962). **e**, Correlation between the aIBFs and cortical LFP slope (example cell). Upper: baseline (Pearson correlation: *R* = 0.3576, ***P = 0.001); lower: ArchT (Pearson correlation: *R* = 0.0362, *P* = 0.7456). **f**, Upper left: Pearson correlation coefficients (*R*) for aIBF–LFP slope correlation (*n* = 7 cells in *n* = 4 mice) during baseline and ArchT condtions. Upper right: box plots for upper-left panel (*R*: 0.21 ± 0.03 versus 0.08 ± 0.02; Student’s paired *t*-test: *P = 0.0141). Lower: *P* values of the Pearson correlations (*n* = 7 cells in *n* = 4 mice) at baseline versus ArchT conditions. Dashed lines: significance levels. **g**, Color plots of LFP traces for low-frequency (lf) and high-frequency (hf) bursts (example cell). Top: baseline. Bottom: ArchT. Colors indicate amplitude. Rows: bursts. 0 ms: first spike in the burst. White asterisks: peaks of individual LFP traces. Red arrow: lag between the LFP peak and the first spike (example burst). Equal numbers of events for all conditions were randomly selected. **h**, STAs for hf and lf bursts during baseline (top) and ArchT (bottom) conditions (*n* = 6 cells in *n* = 4 mice). Dashed line: STA peak for hf bursts, where STA amplitudes were calculated (arrowheads). **i**, STA amplitudes (left) and box plots (right) for hf and lf bursts at the hf peaks during baseline (top) and ArchT (bottom) conditions (*n* = 6 cells in *n* = 4 mice) (baseline: 0.66 ± 0.17 au versus −0.14 ± 0.22 au; Student’s paired *t*-test: *P* = 0.047; ArchT: 0.46 ± 0.11 au versus 0.22 ± 0.12 au, *P* = 0.1657). **j**, Average lag between individual LFP peaks and the first spike (left) and their box plots (right) during baseline (top) and ArchT (bottom) (*n* = 6 cells in *n* = 4 mice) (baseline: −0.18 ± 6.41 ms versus 15.7 ± 4.15 ms; Student’s paired *t*-test: *P* = 0.0056; ArchT: 13.09 ± 7.7 ms versus 8.44 ± 6.48 ms; Student’s paired *t*-test: *P* = 0.1684). Box plots: box shows first to third quartiles; whisker ends indicate minimum and maximum values, x labels the mean. au, arbitrary units; Br., bregma.

**Extended Data Fig. 6:**
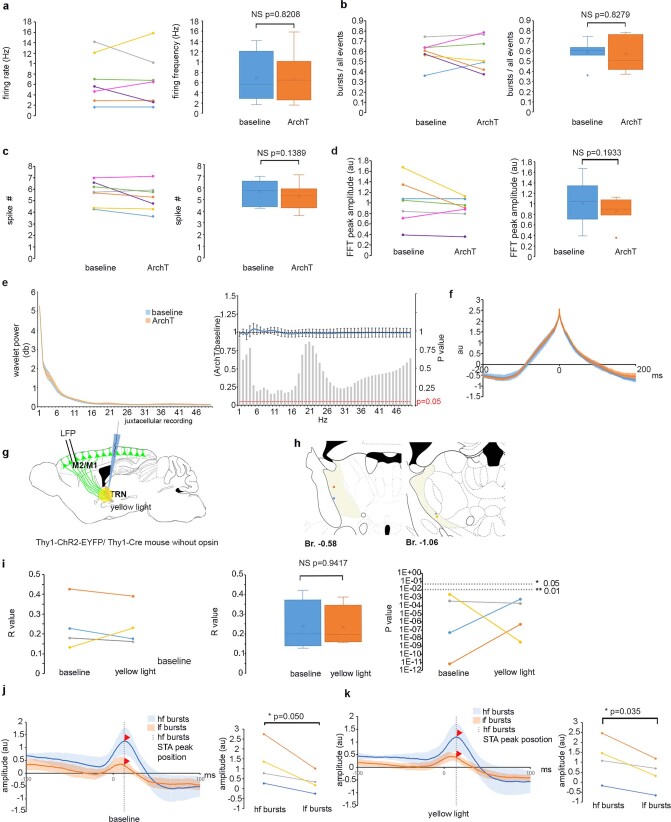
Activity of TRN cells during photoinhibition and control experiments for the L5-TRN optogenetic perturbation protocol. **Related to** Fig. 7. **a**) Left: Firing frequency of TRN cells, baseline vs ArchT. Right: box plots of the same data (6.89 ± 1.76 Hz vs. 6.65 ± 1.9 Hz, Student’s paired sample t-test: NS = 0.8208; Colors of individual cells in a-d match the color code on Fig. 7. N = 7 cells in n = 4 mice. **b**) Same as (a) for fraction of burst from all firing events. (0.59 ± 0.04 Hz vs. 0.58 ± 0.06 Hz, Student’s paired sample t-test: NS p = 0.8279). **c**) Same as (a) for spikes per bursts (5.71 ± 0.39 Hz vs. 5.27 ± 0.44 Hz; Student’s paired sample t-test: NS p = 0.1389). **d**) Left panel: FFT peak amplitude in the 1–4 Hz range baseline vs ArchT. Right: box plots of the same data (1.01 ± 0.16 au vs. 0.87 ± 0.1 au; Student’s paired sample t-test: NS p = 0.1933). **e**) Left: Average wavelet spectra of cortical LFPs, baseline (blue) vs ArChT (orange) conditions. Right: Significance values for each frequency domain comparing baseline and ArchT values (Student’s paired sample t-test). 5% significance level is indicated by red dashed line. Average and ± SEM are indicated. **f**) Average waveforms of fast cortical events baseline (blue) vs ArChT (orange) conditions. Average and ± SEM are indicated. **g**) Schematics of experimental design. **h**) Position of juxtacellularly recorded and filled control TRN cells. Colors of the dots match the colors in (i-k). **i**) Left: Pearson correlation coefficient for the aIBF - LFP slope correlation for baseline vs ArChT conditions in control cells. Middle panel: box plots of the same data (baseline: 0.24 ± 0.06; yellow light: 0.23 ± 0.05); Student’s paired sample t-test: NS p = 0.9417) Right panel: P values of Pearson correlations; baseline vs ArChT conditions. Dahed lines: significance levels. N = 4 cells in n = 2 mice in i, j, k. **j**) Left panel: population average of STA traces during baseline conditions. hf bursts (blue); lf bursts (orange). Average and ±SEM are indicated. Dashed line, STA peak for hf bursts; red arrowheads, STA amplitudes for hf bursts and lf burst. Right: STA amplitudes (hf vs lf) at baseline condition. (hf bursts: 1.28 ± 0.53 au; lf bursts: 0.31 ± 0.26 au; Student’s paired sample t-test: * p = 0.05). **k**) Same as j, during optogenetical perturbation protocol. (STA amplitudes: hf bursts: 1.21 ± 0.55 au; lf bursts: 0.38 ± 0.39 au; Student’s paired sample t-test: *p = 0.035) (yellow light) (right panel). au: arbitrary unit. n = 4 cells in n = 2 mice. au: arbitrary unit. Boxplots: box, first to third quartile; whisker’s ends, minimum and maximum values, x labels mean. au: arbitrary unit.

To test if L5 input is necessary for recruiting TRN neurons during cortical fast LFP transients, we selectively perturbed the activity of L5 terminals in the anterior TRN by locally activating the ArchT inhibitory opsin, which was previously virally delivered to the M1/M2 cortex in Rbp4–Cre and Thy1–Cre transgenic mice (Fig. 7a). We selected TRN cells (Fig. 7b) for further recording by applying 2–3 test pulses (5 seconds each) of yellow laser, which caused a transient, slight decrease in the firing rate (Methods). For data analysis, only cells with significant positive baseline aIBF–LFP slope correlation were used. Position of the recorded cells, and the optic fiber tip and the topography of labeled L5 fibers, were verified post hoc (Fig. 7c). After recording the baseline activity of the cells, we applied yellow light (5 seconds ON and 10 seconds OFF, 60 cycles) to perturb the L5 terminals. Upon optogenetic activation of ArchT, we did not observe a persistent alteration in the firing rate or burst rate of TRN cells (Extended Data Fig. 6a,b). Similarly, the aIBFs (Fig. 7d) and number of spikes per bursts (Extended Data Fig. 6c) were unaffected by the manipulation.

Upon ArchT activation, however, the significant correlation between the aIBF of the bursts and the magnitude of the instantaneous LFP slope observed during baseline activity disappeared in six out of seven cells (Fig. 7e,f). The significant difference between STA peak amplitudes for high-frequency (hf) and low-frequency (lf) bursts observed at baseline activity was also diminished (Fig. 7g–i). During baseline activity, hf bursts occurred significantly earlier than lf bursts relative to the instantaneous LFP peaks. This difference disappeared upon perturbation of L5 inputs (Fig. 7g,j). During ArchT activation, the wavelet and fast Fourier transform (FFT) power spectra of the frontal cortex LFPs (Extended Data Fig. 6d,e) or the average waveform of the fast LFP events did not change (Extended Data Fig. 6f), indicating that the observed effects were not due to an overall change in cortical activity. In control experiments, we did not observe alterations in the correlation between TRN spiking activity and cortical LFP (Extended Data Fig. 6g–k).

These data demonstrate that, although slight perturbation of L5 terminals with ArchT in the TRN did not have major effect on the basic firing properties of the anterior TRN cells, it clearly disrupted the instantaneous correlation between ongoing cortical activity and TRN spiking, indicating a role of the L5 input in controlling the readout of cortical activity by the TRN.

## L5–TRN pathway mediates feed-forward and lateral inhibition

**Fig. 8:**
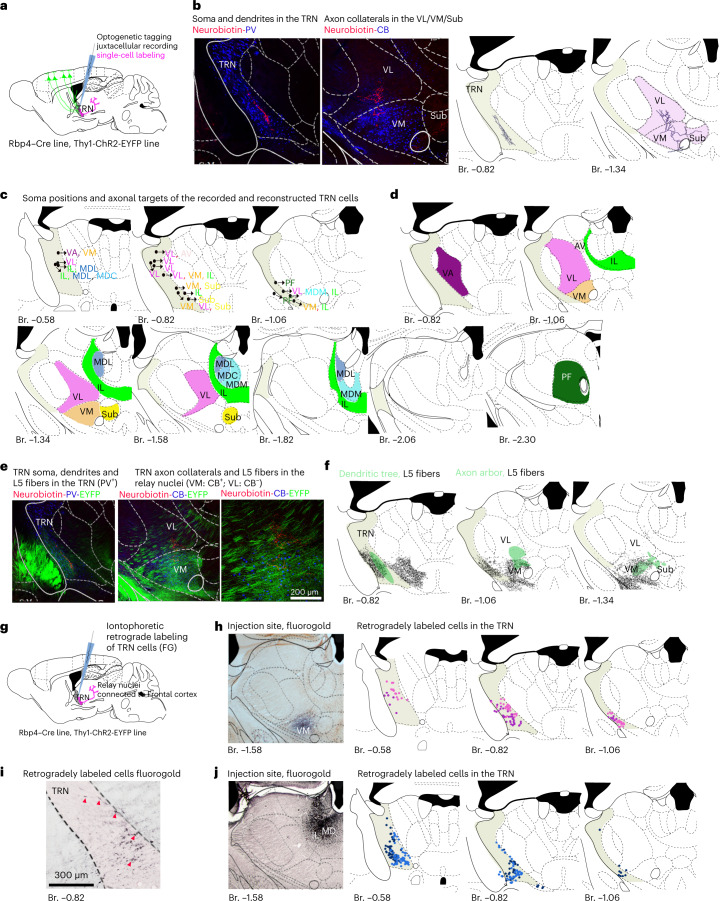
TRN cells receiving L5 input target frontal-cortex-related relay nuclei. **a**, Experimental design. **b**, Example neurobiotin-filled neuron. Left panel: cell body and dendrites in the TRN (PV^+^). Middle left: axon arbor in the VM (CB^+^) and VL (CB^−^). Middle right: reconstructed soma and dendritic tree. Right: reconstructed axon arbor (target nuclei are labeled with pale red). **c**, Soma position of reconstructed neurons (*n* = 18 in *n* = 14 mice, from which *n* = 7 Thy1-ChR2-EYP and *n* = 7 Rbp4–Cre). Axon arborization zones are indicated by abbreviations of the target nuclei. Font colors match colors on **d**. Primary target nuclei are labeled in bold. **d**, Target nuclei of the neurons shown in **c**. For individual axon arbors, see Extended Data Fig. 7. **e**, Left: example optogenetically tagged and neurobiotin-filled neuron from the Rbp4–Cre mice surrounded by labeled L5 fibers (EYFP^+^) originating from M1/M2. Middle: axon arbor of the same neuron and the L5 fibers from M1/M2. Right panel: higher magnification for the middle panel. **f**, Schematics depiction of **e**. Black, L5 collaterals; green, dendritic (left) and axon arbor (middle and right) of the TRN neuron. For more examples, see Extended Data Fig. 8. **g**, Experimental design. **h**, Left: injection site in the VM. Middle and right: position of retrogradely labeled somata in the TRN (*n* = 2 mice, labeled with different shades). **i**, Retrogradely labeled cells in the anterior TRN after retrograde tracer injection to the MD. Red arrowheads, retrogradely labeled TRN cells. **j**, Left: injection site in the MD. Middle and right: position of retrogradely labeled somata in the TRN (*n* = 2 mice, labeled with different shades). AV, anteroventral nucleus; Br., bregma; FG, fluorogold. VM TRN is labeled with gray.

**Extended Data Fig. 7:**
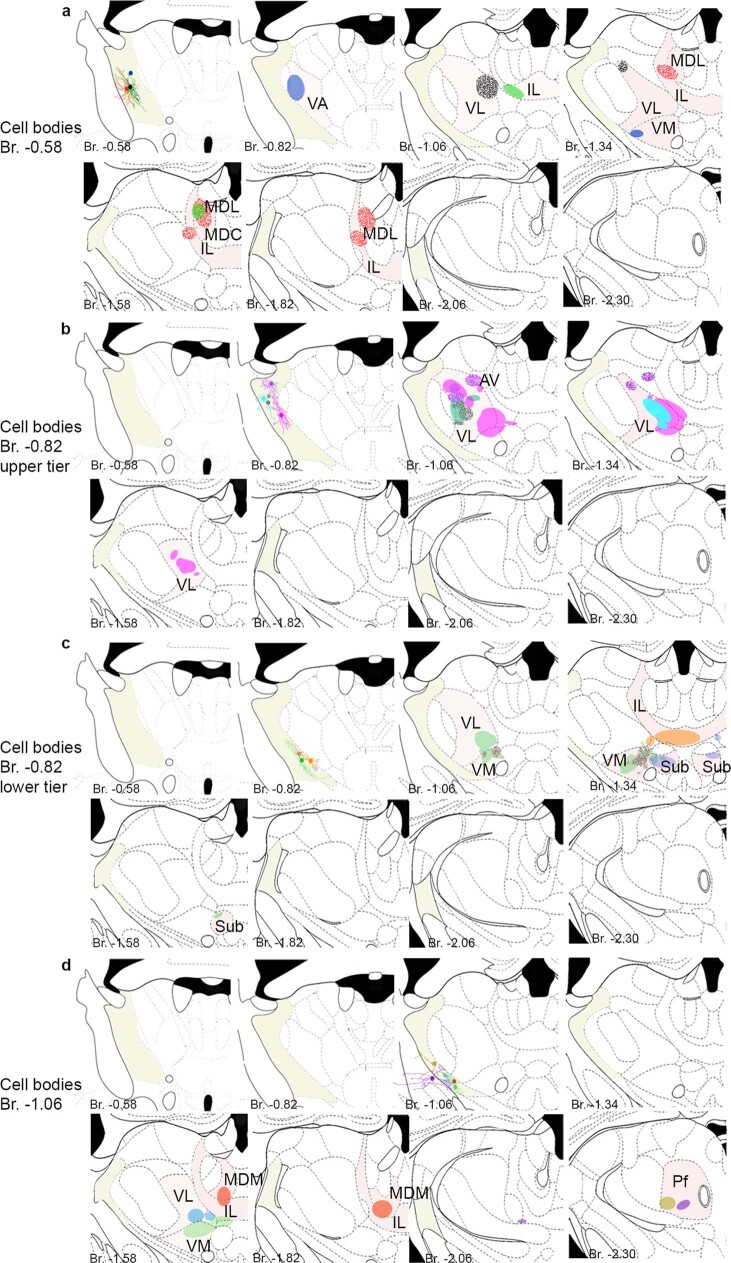
Reconstructed dendritic trees, cell body positions and axonal target zone of juxtacellularly recorded and labelled TRN cells optogenetically tagged by their L5 afferents. **a**) Reconstruction of 4 TRN cells with cell bodies situated at the Br. −0.58 coronal level in eight antero-posterior levels. Dots of different colors within the TRN indicate cell body position. When dendritic trees were filled well enough, it is labelled with the same color as the soma. Axonal target zones in the relay nuclei are indicated with ellipses. Colors of somatic labelling match those of the projection zone. TRN is labelled with pale yellow, target relay nuclei are labelled with pale red. TRN: thalamic reticular nucleus; VA: ventral anterior nucleus; VL: ventral lateral nucleus; IL: intralaminar complex; MDL: mediodorsal nucleus, lateral part; MDC: mediodorsal nucleus, central part; VM: ventral medial nucleus; Br: Bregma. **b**) Same as (a) for 5 TRN cells with cell bodies at the Br. −0.82 coronal level, in the upper TRN tier. VL: ventral lateral nucleus; AV: anteroventral nucleus; Br: Bregma. **c**) Same as (a) for 4 TRN cells with cell bodies at the Br. −0.82 coronal level in the lower TRN tier. VL: ventral lateral nucleus; VM: ventral medial nucleus; IL: intralaminar complex; Sub: submedius nucleus; Br: Bregma. **d**) Same as (a) for 5 TRN cells with cell bodies at the Br. −1.06 coronal level. VL: ventral lateral nucleus; VM: ventral medial nucleus; IL: intralaminar complex; MDM: mediodorsal nucleus, medial part; Pf: parafascicular nucleus Br: Bregma.

**Extended Data Fig. 8:**
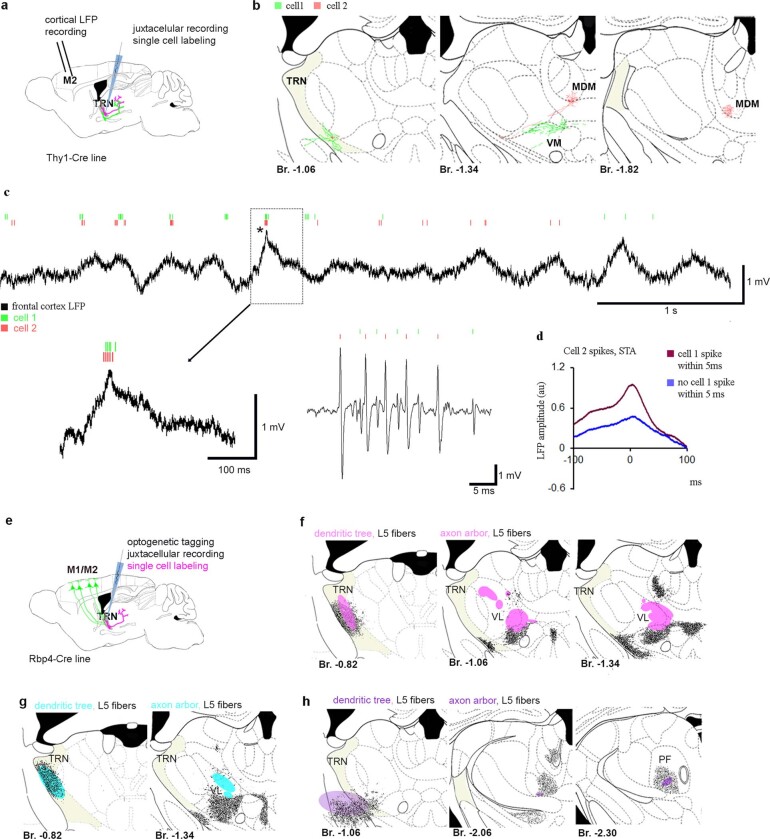
TRN neurons projecting to different relay nuclei synchronize their activity during sharp cortical events and morphological bases for feed forward and lateral inhibition in the L5-TRN pathway. (**a**) Schematics of experimental design. (**b**) Left panel: Cell bodies and dendrites of the two TRN cells. For cell one the dendritic tree was only partially labelled. Middle and right panels: Axon arbour of the two cells in the VM and MDM relay nuclei respectively. TRN, thalamic reticular nucleus; VM, ventral medial nucleus; MDM, mediodorsal nucleus, medial part; Br, Bregma. (K). (**c**) Example of firing activity of the two simultaneously recorded TRN cells and the simultaneously recorded frontal cortical LFP activity. Fast LFP event is labelled with asterix, and magnified in the islet below (lower left panel). Lower right panel: raw unit recording under the fast event. (**d**) Spikes of cell1 were sorted in two groups regarding if they were preceded or followed by a cell2 spike within a 5 ms time window. Spike triggered cortical LFP averages (STAs) were calculated for the two groups. When the two cells fired synchronously (within 5 ms) the STA had higher peak amplitude and larger slope around the spike compared to the STA of spikes when the two cells were less synchronous. au: arbitrary unit. (**e**) Schematics of experimental design. (**f**) Schematic representation of the dendritic (left panel) and axon (right panel) arbors (magenta) of an example neurobiotin filled TRN neuron optogenetically tagged from L5. Virus labelled L5 fibers are shown in black. TRN: thalamic reticular nucleus; VL: ventral lateral nucleus; Br: Bregma. (**g**) Same as (f) for another TRN cell. (**h**) Same as (f). In this case, L5 and TRN target zones overlap, implying feed forward inhibition. Dendritic tree of this particular TRN neuron extends to the internal capsule. TRN: thalamic reticular nucleus; PF: parafascicular nucleus; Br: Bregma.

What are the thalamic targets of the TRN cells conveying the integrated activity of L5 neurons? To study this, we reconstructed the complete axon arbor of neurobiotin-filled TRN cells optogenetically tagged via their L5 inputs (Figs. 1o, 6b, 7b and 8a–f and Extended Data Figs. 6h and 7). All TRN neurons targeted thalamic nuclei known to be connected with the frontal cortex^2^. These include the ventral lateral nucleus (VL): *n* = 8; ventral anterior nucleus (VA): *n* = 1; ventral medial nucleus (VM): *n* = 5; intralaminar complex (IL): *n* = 6; parafascicular nucleus (Pf): *n* = 2; mediodorsal nucleus, lateral part (MDL): *n* = 2; mediodorsal nucleus, central part (MDC): *n* = 1; mediodorsal nucleus, medial part (MDM): *n* = 1; and submedius nucleus (Sub): *n* = 3 (Fig. 8c–d). Interestingly, nine of the 18 cells had more than one target nucleus (VA-VM: *n* = 1; VM-Sub: *n* = 1; VM-VL-Sub: *n* = 1; VL-VM-IL: *n* = 1; VM-IL: *n* = 1; MD-IL: *n* = 3; and VL-AV: *n* = 1). Although there was a loose topography regarding the position of the cell bodies and the axonal targets, TRN neurons with different targets could be found intermingled (Fig. 8c and Extended Data Fig. 8a–d).

In accordance with these data, retrograde labeling experiments confirmed that TRN cells targeting frontal-cortex-related relay nuclei (VM and MD) are in the anterior, L5-recipient part of TRN (Fig. 8g–j).

To resolve whether the TRN transmits feed-forward or lateral inhibition, we reconstructed the axon arbors of TRN cells in the thalamus together with the L5 fibers through which they were activated in the Rbp4–Cre mice (*n* = 5 cells in *n* = 4 mice). We examined whether TRN axons are inside (indicating feed-forward inhibition) or outside (lateral inhibition) the labeled cortical terminal field in the thalamus (Fig. 8e,f and Extended Data Fig. 8e–h).

In three cases, a large proportion of the TRN axon arbor was outside the termination zone of the cortical L5 fibers, implying that lateral inhibition can be substantial in the L5–TRN pathway. In all three cases, the L5 axons were confined to VM, whereas TRN axons innervated VL. In the other two cases (VM and Pf nuclei), TRN axonal targets were completely within the L5 zone (*n* = 2 cells), indicating feed-forward inhibition in case of these neurons.

In one of the in vivo electrophysiology experiments, two TRN cells could be recorded and filled simultaneously in the anterior TRN (Extended Data Fig. 8a). The cell bodies were in close vicinity of each other (within 100 µm), but the two TRN cells targeted two different relay nuclei (MD and VM, respectively) (Extended Data Fig. 8b). Upon fast cortical events, the firing of the two cells became tightly correlated (Extended Data Fig. 8c). STA for the spikes that were paired with a spike of the other cell within a 5-ms time window had a higher amplitude and narrower peak than the STA of the spikes that were less synchronous with the activity of the other cell (Extended Data Fig. 8d).

These data show that L5 neurons in one cortical location can have widespread inhibitory action in the thalamus via their connection with TRN and suggest that synchronous cortical events can synchronize the activity of multiple relay nuclei via the L5–TRN output.

## Discussion

Here we described and characterized a specific and topographically organized pathway that originates from frontal cortex L5 PT cells and that selectively targets the anterior TRN. The data showed that, via integrating multiple L5 inputs, the exact spike output of the TRN cells provide a sensitive measure of synchronous cortical activity. The output of frontal L5-driven TRN activity reached thalamic regions connected to the frontal cortices. These data indicate that cortical control of thalamic activity is region-specific and that frontal L5–TRN projection can be instrumental in sculpting thalamic activity in widespread frontal cortex functions involving synchronous cortical firing.

Until recently, the organization of corticothalamic connections was considered canonical^4^: all cortical regions were reported to send both L6 and L5 projections to the thalamus, but only L6 corticothalamic axons have been shown to establish synaptic connections in the TRN. Indirect evidence from previous reports indicated the presence of L5 synaptic input in the TRN^33,34^. However, none of these works provided direct, conclusive evidence for a monosynaptic L5–TRN connection, cell type specificity or regional variability of its source^35^ and their physiological features. Using both morphological and physiological methods, we clearly demonstrate monosynaptic connection selectively from L5 PT cells (Fig. 2) of the frontal cortex to the TRN and, thereby, provide direct evidence for qualitative differences between cortical regions regarding the way they recruit intrathalamic inhibition (Fig. 1). Although the main body of our experiments focused on the L5 inputs arising from the M1/M2 cortical regions, viral tracing experiments clearly demonstrated the presence of the L5–TRN projection from other frontal cortex areas, such as Prl, Cg and orbitofrontal cortex (Extended Data Figs. 1 and 2).

We demonstrated that, along the specific L5–TRN projection from the frontal cortex, the canonical L6–TRN input forms a highly convergent, parallel pathway in the anterior TRN (Fig. 3a–c and Extended Data Fig. 2g–j). The two cortex–TRN pathways have distinct morphological and functional properties (Figs. 3 and 4). The difference in the organization of L6–TRN and L5–TRN pathways is similar to those of the L6–thalamic and L5–thalamic pathways^36^, confirming complementary roles of the two projections. The larger volume of L5–TRN boutons can be largely attributed to the presence of multiple mitochondria, suggesting intensive synaptic activity^37^. The diameter of the dendrites negatively correlates with the distance from the soma. Thus, the larger diameter of the post-synaptic dendrites in the case of the L5–TRN synapses suggests more proximal and more effective synaptic connection. The presence of spine synapses, the complex post-synaptic density (PSD) morphology (Fig. 3) and the elevated NMDA/AMPA ratio (Fig. 4) may indicate a higher potential for synaptic plasticity at the L5–TRN synapses^32,38^.

Our data show that individual TRN cells effectively integrate the activity of multiple pre-synaptic L5 cells. Although individual L5 EPSCs were small in vitro (Fig. 4), optogenetically activated L5 neurons could reliably fire TRN cells in vivo (Fig. 1). The response probability and the exact spike output of the optogenetically evoked TRN responses in vivo correlated with the size of recruited L5 population activity (Fig. 6 and Extended Data Fig. 3). Moreover, during spontaneous activity, the firing pattern of 61.3% of the anterior TRN neurons provided a gradual readout of the magnitude of synchronous cortical activity, which could be detected in the LFP recordings as fast, high-amplitude transients (Fig. 6). Although single spikes were uncorrelated with the cortical LFP, bursts were specifically coupled to the fast cortical transients, and the burst properties of the TRN neurons significantly correlated with the magnitude of the instantaneous LFP slope (Fig. 6). Bursts have been described in both awake and sleep states in the anterior TRN^39^. Although bursts are often viewed as stereotypical intrinsic all-or-none events mediated by T-type calcium channels, experimental evidence shows considerable variety in the TRN burst properties, which can correlate with, for example, the complexity of the behavior^39^ or with the parameters of the corticothalamic oscillations^6^. Furthermore, Kepecs et al.^40^ showed that bursts tend to occur at the positive slope of the synaptic input signals and that burst properties can code the magnitude of the signal slope. Based on this, we propose that there is a strong synaptic component contributing to the burst generation in the anterior TRN and that the exact spike output and burst pattern of TRN neurons will code the level of synchronous L5 activity in the cortex.

Optogenetic perturbation of L5 fibers in the TRN further confirmed the critical role of L5–TRN input to transmit fast changes in cortical activity to anterior TRN cells (Fig. 7). Upon ArchT-mediated disruption of L5–TRN inputs, the correlation between TRN burst properties and the instantaneous cortical LFP activity was disturbed. Earlier work showed that ArchT activation in pre-synaptic terminals does not result in a clear inhibition of synaptic transmission but, rather, in a mixture of decreased probability of action-potential-evoked release and increased probability of spontaneous synaptic release^41^. In line with this, we did not observe long-term decrease in firing rates of the TRN neurons upon sustained ArchT activation of their L5 inputs. Decoupling of pre-synaptic activity and precise transmitter release in the L5 terminals via ArchT, however, was sufficient to disrupt the correlation between the cortical and the TRN activity. These data show that precise and effective integration of L5 output is required to convert cortical activity to a TRN action potential output pattern.

What might be the significance of the exact TRN spike output for the post-synaptic thalamocortical neurons? TRN bursts can increase the inhibitory post-synaptic current (IPSC) magnitude in the post-synaptic relay cells compared to single TRN action potentials^42^. Mechanism of burst IPSCs is unlike that of single action potentials because GABA released during bursts can recruit non-synaptic GABA_A_ receptors, which results in a significantly different inhibitory charge and kinetics^43,44^. During slow-wave sleep, the exact number of spikes/TRN burst changes stereotypically during sleep spindles and cycle-by-cycle reduction in spike/burst was suggested to be a major determinant of terminating this sleep transient^6^. Thus, the impact of different TRN spike patterns on relay cell firing and signal integration is clearly significant but certainly needs further investigation.

Synchronous activity can arise locally or from multiple regions of the frontal cortex. In our viral tracing experiments, L5 axons from the neighboring frontal cortex territories showed clear segregation (Fig. 5). Our data were in good agreement with the single-cell reconstruction data from the Mouse Light Neuron Browser and with a paper^30^ that reported loose but clear topography in the cingulate cortex–TRN pathway. Topographical termination of frontal L5 fibers in the TRN suggested that TRN cells at a given spatial position may integrate inputs from a relatively narrow cortical territory. The extensive dendritic tree of the L5-driven TRN neurons (Figs. 1 and 6–8), however, may extend across multiple termination zones, so TRN cells could integrate more-global synchronous cortical activity. Indeed, our experiments demonstrated that, although TRN cells showed preferential activation from specific frontal cortex areas, they could be activated from multiple frontal cortex territories (Fig. 5).

Tracking the axons of L5-driven TRN cells clearly showed that, through their TRN collaterals, L5 neurons of the frontal cortex can have widespread inhibitory action in large thalamic regions related to the frontal cortex (Fig. 8). Our experiments revealed an anatomical basis for feed-forward inhibition in the VM and Pf nuclei. In contrast, several L5-recipient TRN cells innervated the VL nucleus, which, as a first-order nucleus, does not receive L5 input from the frontal cortex^45^ (Fig. 8 and Extended Data Fig. 7), indicating lateral inhibition and cross-modal interactions in the case of VL. L5-driven TRN cells frequently innervated multiple thalamic nuclei (a rare feature of TRN cells^46^), and TRN cells targeting different thalamic nuclei displayed correlated activity during fast cortical transients (Extended Data Fig. 8). This suggests that synchronous cortical events can synchronize the activity of multiple relay nuclei via the L5–TRN output.

Feed-forward and lateral inhibition are fundamental mechanisms of neuronal circuits, which, among other factors, are pivotal for gain control^3^, synchronization of high-frequency activity^47^, frequency-dependent signal transfer^48^ or receptive field tuning^49^. Our data suggest that corticothalamic L5 pathways are heterogeneous in this respect. We show here that, in contrast to sensory corticothalamic information transfer, the vast array of frontal cortex functions use an additional, powerful form GABAergic mechanism at the level of thalamus. Because frontal cortex is implicated in diverse neurological conditions (for example, Parkinson’s disease, epilepsy and chronic pain), and thalamic neurons respond robustly to TRN inhibition, the frontal L5–TRN projection characterized here may potentially play a critical role in establishing and/or maintaining these pathological conditions.

## Methods

### Animals

Animal use was approved by the Animal Welfare Committee of the Institute of Experimental Medicine in Budapest, Hungary, in accordance with the regulations of the European Community’s Council Directive of 24 November 1986 (86/609/EEC). Experiments were approved by the National Animal Research Authorities of Hungary (PE/EA/877-7/2020). Mice were maintained on a 12-hour light/dark cycle, and food and water were provided ad libitum. All mice were healthy with no obvious behavioral phenotypes. For all mouse studies, adult male mice (6–14 weeks of age) were used. Mice were randomly allocated to experimental groups: C57Bl/6J-Tg (Rbp4–Cre) (stock Tg(Rbp4-cre)KL100Gsat/Mmucd, ID: MMRRC_031125-UCD)^50^, C57Bl/6J-Tg (Thy1-ChR2-YFP) (JAX stock 007612)^51^, FVB/AntFx-Tg (Thy1–Cre) (JAX stock 006143)^52^ and Bl6Fx-Tg (Ntsr1–Cre) (stock Tg(Ntsr1-cre)GN209Gsat/Mmucd, ID: MMRRC_030780-UCD)^50^.

### Surgery

Mice were anesthetized with an intraperitoneal injection of ketamine–xylazine (ketamine, 83 mg kg^−1^, Produlab Pharma, 07/01/2302; xylazine, 3.3 mg kg^−1^, Produlab Pharma, 07/03/2303) and placed inside a stereotactic apparatus. Depth of anaesthesia was monitored throughout the surgery, and additional dose (ketamine, 28 mg kg^−1^; xylazine, 1.1 mg kg^−1^) of anaesthetic was applied intramuscularly if necessary.

#### Viral injections

Virus injections were performed on adult male Rbp4–Cre (*n* = 44), Thy1–Cre (*n* = 3) and Ntsr1–Cre (*n* = 9) mice. AAV5.EF1a.DIO.hChR2(H134R)-eYFP.WPRE.hGH (based on Addgene plasmid 20298, UNC Vector Core), AAV5.EF1.dflox.hChR2(H134R)-mCherry.WPRE.hGH (based on Addgene plasmid 20297, UNC Vector Core), AAV5.CAG.Flex.ArchT-GFP (based on Addgene plasmid 28307, UNC Vector Core) and AAV.DFO.ChR2-eYFP^53^ viruses were injected in the right-side neocortex or brainstem (200 nl, 1 nl s^−1^) using borosilicate glass capillaries. Stereotaxic coordinates were the following (AP and ML taken from bregma, DV taken from the brain surface, in mm):

Cortical injections resulting in L5–TRN collaterals: M2: AP +2, ML +0.5, DV −0.7; (in case of double injections to M2, M2 anterior: AP: +2.5, ML +0.7, DV, −0.7; M2 posterior: AP +1.5, ML +0.7, DV −0.7); M1: AP +2, ML +2, DV −0.7; FrA: AP +2.7, ML +1.5, DV −0.7; orbitofrontal cortex: AP +2.2, ML +1.2, DV +2; medial prefrontal cortex (mPFC) anterior: AP +2, ML +0.4, DV −1.2; mPFC posterior: AP −1, ML +0.5, DV +0.7 or AP −2, ML −0.5, DV −0.7.

Cortical injections resulting in no L5–TRN: S1: AP +1.1, ML +2.6, DV −1; S1BF: AP −1.3, ML +3, DV −0.7; S2: AP −0.1, ML +3.5, DV −1.7; GI/DI: AP +0.1, ML +3.6, DV −2.3.

Brainstem (pontine reticular nucleus, oral part (PnO)): AP −4.4, ML 0.8, DV −4.2.

#### Tracer injections

Retrograde tracer injections were performed on adult wild-type littermates of Rbp4–Cre mice (*n* = 4). Fluorogold (Sigma-Aldrich, AB153-I) was injected iontophoretically (0.5 μA; 2-second ON/OFF period, 10-minute duration) in the right side of the thalamus using borosilicate glass capillaries. Stereotaxic coordinates were the following: VM: AP −1.3, ML 0.8, DV −4.2; MD: AP −1.3, ML 0.5, DV −2.9.

### Histology

Mice were perfused with 4% paraformaldehyde (TAAB Laboratory, P001) and 0.1% glutaraldehyde (Electron Microscopy Sciences, 16210) in PB (0.1 M). Coronal sections (50-μm-thick) were cut with a vibratome. To permeabilize the membranes, sections were incubated in sucrose (30%) overnight, followed by freeze–thawing over liquid nitrogen. Fluorogold was visualized with a rabbit anti-Fluorogold antibody (1:10,000, Millipore, AB153-I), followed by biotinylated goat anti-rabbit (1:300, Vector Laboratories, BA-1000) and avidin biotinylated horseradish peroxidase complex (ABC, 1:300, Vector Laboratories, PK-4000). Nickel-intensified 3,3′-diaminobenzidine (DABNi, bluish-black reaction product, DAB: Sigma-Aldrich, D5637) was used as a chromogen. EYFP and mCherry fluorescent labels were intensified via chicken anti-GFP antibody (1:5,000, Thermo Fisher Scientific, A10262), followed by goat anti-chicken-Alexa 488 antibody (1:500, Thermo Fisher Scientific, A-11039) and rabbit anti-mCherry antibody (1:3,000, BioVision, 5993-100), followed by donkey anti-rabbit-Cy3 antibody (1:500, Jackson, AB_2307443), respectively. Neurobiotin content of juxtacellularly labeled cells was visualized by Cy3-streptavidin (1:500, Jackson, 434315). For reconstructing neurobiotin-labeled TRN cells, DABNi staining was developed applying avidin biotinylated horseradish peroxidase complex (see above). Cortical terminals were labeled by VGLUT1 (rabbit anti-VGLUT1 antibody, 1:10,000, Millipore, ABN1647; donkey anti-rabbit Cy3 antibody, 1:500, Jackson). Subcortical glutamatergic terminals were labeled by VGLUT2 (rabbit anti-VGLUT2 antibody, 1:10,000, Synaptic Systems, 135404; donkey anti-rabbit Cy3 antibody, 1:500, Jackson). TRN and higher-order relay nuclei were stained with PV (mouse anti-PV antibody, 1:2,000, Sigma-Aldrich, P3088; donkey anti-mouse-Cy5, 1:500, Jackson, AB_2340820) and CB (rabbit anti-CB antibody, 1:2,000, Swant, CB-38a; donkey anti-rabbit Cy5, 1:500, Jackson, AB_2340607), respectively. Results were obtained with a Zeiss Axioplan 2 fluorescent microscope and a digital camera (Olympus, DP70), with a Zeiss Axio Imager M1 microscope coupled to an Axiocam HrC digital camera or with a Nikon AR1 confocal microscope.

### Electron microscopy

M1/M2 cortices of adult male Rbp4–Cre (*n* = 2) or Ntsr1–Cre (*n* = 2) mice were injected with AAV5-EF1a-DIO-ChR2-EYFP virus. Two weeks after surgery, mice were perfused with 4% paraformaldehyde and 0.1% glutaraldehyde in PB (0.1 M). Coronal sections (50-μm-thick) were cut with a vibratome. To permeabilize the membranes, sections were incubated in sucrose (30%) overnight, followed by freeze–thawing over liquid nitrogen. For visualizing labeled fibers, sections were incubated with rabbit anti-GFP antibody (1:2,000, Thermo Fisher Scientific, A-11122) overnight, followed by biotinylated b-SP donkey anti-rabbit antibody (1:300, Jackson, AB_2340593). Sections were incubated with ABC complex (1:300) for 2 hours. Staining was developed with DABNi. Sections were treated with OsO_4_, dehydrated in ethanol and propylene oxide and embedded in Durcupan (Sigma-Aldrich, 44610). During dehydration, sections were treated with 1% uranyl acetate in 70% ethanol. Selected blocks were re-embedded, and 60-nm-thick ultrathin sections were cut with an ultramicrotome. Sections were mounted on copper grids. Electron micrographs were taken with a MegaView digital camera running on a Hitachi 7100 electron microscope. Reconstruct software was used for three-dimensional (3D) reconstruction. Cell membranes of pre-synaptic and post-synaptic structures, PSDs and mitochondria in the boutons were reconstructed. For measurements, Fiji software was used on raw pictures. Minor diameters of post-synaptic dendrites were measured in three non-consecutive sections and averaged. Bouton volume, PSD area and number of mitochondria were calculated in boutons where consecutive sections containing the full extent of the given structure were preserved and the ultrastructure^54^ of the tissue was appropriate.

### In vivo electrophysiology

#### Anesthesia and surgery

Adult male Thy1-ChR2-EYFP (*n* = 20), Rbp4–Cre (*n* = 10) and Thy1–Cre (*n* = 3) mice were used for the experiments. In the case of Rbp4–Cre and Thy1–Cre mice, electrophysiology experiments were carried out 2–4 weeks after the virus (AAV5.EF1a.DIO.hChR2(H134R)-eYFP.WPRE.hGH or AAV5.CAG.Flex.ArchT-GFP) injection to M2 and M1 (200 nl and 200 nl). Surgeries and experiments were done under ketamine–xylazine anesthesia. Mice received intraperitoneal injection of ketamine (50 mg kg^−1^) and xylazine (4 mg kg^−1^). For *n* = 3 mice, we used the doses used at surgeries for virus or tracer injections (ketamine, 83 mg kg^−1^; xylazine, 3.3 mg kg^−1^). This higher dose, however, induced a deep anaesthesia characterized by a frontal cortex LFP dominated by a regular, slow (1–4 Hz) component and by a predominantly tonic firing of TRN neurons. For this reason, mice receiving this higher dose of ketamine–xylazine were excluded from the analyses examining cortical LFP–TRN firing correlation. For optogenetic perturbation experiments using ArchT, and for their control experiments, 36 mg kg^−1^ of ketamine and 2.9 mg kg^−1^ of xylazine were used. For maintenance of the anesthesia, intramuscular injection of ketamine–xylazine (1/3 of the initial amount) was given every 30–50 minutes during the duration of the experiments.

#### In vivo juxtacellular recording and labeling and LFP recording

Bipolar LFP electrodes (FHC, resistance ~1 MΩ) were placed into the frontal cortex (AP: +2.5 mm, ML: 1 mm from bregma). The recorded signal was amplified, band-pass filtered from 0.16 Hz to 5 kHz (Supertech) and digitized at 20 kHz (micro 1401 mkii, CED). Cortical L5, TRN or VM single-unit activity was recorded by glass microelectrodes (in vivo impedance of 20–40 MΩ) pulled from borosilicate glass capillaries (1.5-mm outer diameter, 0.75-mm or 0.86-mm inner diameter, Sutter Instrument) and filled with 0.5 M K^+^-acetate and 2% neurobiotin (Vector Laboratories, SP-1120). Electrodes were lowered by a micromanipulator (Scientifica) to the target area (cortex: AP +2, ML +0.5, DV −0.5 to 0.9; TRN: AP −0.7, ML 1.6, DV −2.3 to 4; VM: AP −1.3, ML +0.9, DV −3.8 to 4.3; AP and ML taken from bregma, DV taken from the brain surface, in mm). Neuronal signals were amplified by a DC amplifier (Axoclamp 2B, Axon Instruments/Molecular Devices), further amplified and filtered between 0.16 Hz and 5 kHz by a signal conditioner (LinearAmp, Supertech) and recorded by Spike2 7.0 (CED). Juxtacellular labeling of the recorded neurons was done as described previously^55^. For histological analysis, see the Methods ‘Histology’ section.

### In vivo optogenetics

For optogenetic activation, adult male Thy1-ChR2-EYFP (*n* = 20) and Rbp4–Cre (*n* = 7) mice were used. In the case of Rbp4–Cre mice, AAV5.EF1a.DIO.hChR2(H134R)-eYFP.WPRE.hGH virus was injected (see the Methods ‘Surgery’ section). For details of juxtacellular and LFP recordings, see the Methods ‘In vivo electrophysiology’ section. The skull was thinned above the M2/M1 of the right hemisphere, where the optic fiber (100 µm, 0.22 NA) was positioned (M2: AP +2 mm, ML +0.5 mm; M1: AP +2 mm, ML +2 mm, from bregma). Laser beam was generated by a 473-nm DPSS laser (Laserglow Technologies). Laser power at the optic fiber tip was measured before and after each experiment with a photometer (Thorlabs). In addition, laser power was monitored throughout the experiment via a photometer (Thorlabs) built in the laser path. L5 cells and TRN or VM cells targeted by labeled L5 fibers were optogenetically tagged by test laser pulses (5 ms, 10 mW). After recording the baseline activity for 300 seconds, five stimulus trains of 10×1-Hz pulses (5 ms) generated by Spike2 7.0 software (CED) were applied in case of TRN and VM cells.

For optogenetic perturbation of L5 to TRN inputs, adult male Thy1–Cre (*n* = 2) and Rbp4–Cre (*n* = 2) mice were injected with AAV5.CAG.Flex.ArchT-GFP virus (see the Methods ‘Surgery’ section). For control experiments (*n* = 2 mice), Thy1-ChR2-EYFP and Thy1–Cre mice were used. Juxtacellular recording and cortical LFP recording were carried out as described in the Methods ‘In vivo electrophysiology’ section. Custom-made mirror tip optic fibers (200 µm, 0.37 NA, Doric) were lowered to the target area in the TRN (AP −0.5, ML 0.8, DV −4; AP and ML taken from bregma, DV taken from the brain surface, in mm) in 20° angle, with the mirror facing toward the TRN, preventing the light from spreading to the neighboring relay nuclei. The laser beam was generated by a 589–594-nm DPSS laser (Laserglow Technologies). Laser power at the tip of the optic fiber was measured before and after each experiment with a photometer (Thorlabs). TRN cells targeted by labeled L5 fibers were found via optogenetic tagging by test laser pulses (5 seconds, 10 mW). A transient drop in in the firing frequency of the targeted cells could be observed. In control experiments, cells near the optic fiber tip were found by optogenetic tagging with 5-ms, 5-mW test pulses (473 nm, Thy1-ChR2-EYFP mouse) or post hoc by measuring the distance between the position of the soma and the optic fiber tip (<200 µm, Thy1–Cre mouse). After recording the baseline activity for 300 seconds, 60 cycles of 5-second-long 589–594-nm laser pulses, followed by a 10-second laser OFF period, were applied. Position of the optic fiber, labeled L5 terminals and TRN somas were verified for each experiment after histological processing of the brains (see the Methods ‘Surgery’ section).

### In vitro electrophysiology

#### Brain slice preparation

Rbp4–Cre (*n* = 10) and Ntsr1–Cre (*n* = 4) adult male mice were sacrificed 3–4 weeks after viral injection. Mice were anesthetized with isoflurane and their brains quickly extracted. Acute 300-µm-thick sections were sliced using a sliding vibratome (Histocom) while submerged in ice-cold oxygenated sucrose solution (which contained, in mM: 66 NaCl, 2.5 KCl, 1.25 NaH_2_PO_4_, 26 NaHCO_3_, 105 D(+)-saccharose, 27 D(+)-glucose, 1.7 L(+)-ascorbic acid, 0.5 CaCl_2_ and 7 MgCl_2_). Slices were stored in a recovery solution (in mM: 131 NaCl, 2.5 KCl, 1.25 NaH_2_PO_4_, 26 NaHCO_3_, 20 D(+)-glucose, 1.7 L(+)-ascorbic acid, 2 CaCl_2_, 1.2 MgCl_2_, 3 myo-inositol and 2 pyruvate) at 35 °C for 30 minutes and then at room temperature for 30 minutes before recording.

#### Whole-cell patch-clamp recording and optogenetic stimulation

Recording extracellular solution (containing, in mM: 131 NaCl, 2.5 KCl, 1.25 NaH_2_PO_4_, 26 NaHCO_3_, 20 D(+)-glucose, 1.7 L(+)-ascorbic acid, 2 CaCl_2_ and 1.2 MgCl_2_) was supplemented with 0.1 picrotoxin and 0.01 glycine when appropriate (Fig. 4e), maintained at room temperature, constantly oxygenated and perfused in the recording chamber. Borosilicate glass pipettes (TW150F-4, World Precision Instruments) were filled with an intracellular solution containing (in mM): 140 K-gluconate, 10 HEPES, 10 KCl, 0.1 EGTA, 10 phosphocreatine, 4 Mg-ATP, 0.4 Na-GTP, pH 7.3, 290–305 mOsm, supplemented with ∼2 mg ml^−1^ of neurobiotin and showed a pipette resistance ranging from 2.5 MΩ to 5 MΩ for most recordings. NMDAR-mediated currents (Fig. 4e) were recorded using intracellular solution containing (in mM): 127 Cs-gluconate, 10 HEPES, 2 Cs-BAPTA, 6 MgCl_2_, 10 phosphocreatine, 2 Mg-ATP, 0.4 Na-GTP, 2 QX314-Cl, supplemented with ~2 mg ml^−1^ of neurobiotin, pH 7.3, 290–305 mOsm.

Whole-cell patch-clamp recordings were performed as previously described (Vantomme et al.^11^). In brief, passive cellular properties were measured immediately after gaining whole cell access while holding the TRN cell at −60 mV. L5 and L6 afferents were activated using whole-field blue LED (Cairn Research) stimulation (455 nm, duration: 0.1–1 ms, maximal light intensity 3.5 mW, 0.16 mW/mm^2^) during voltage-clamp recordings. The properties of the EPSCs were measured at −60 mV. Paired-pulse stimulation at 1, 2, 5, 10 and 20 Hz were used to measure the short-term properties of L5–TRN and L6–TRN synapses. AMPA receptor-mediated component was measured at −60 mV and blocked using 40 µM DNQX. Once fully blocked, the cell was clamped at +40 mV to record the NMDA-receptor-mediated component. D,L-APV (100 μM) was then used to fully block the evoked response.

### Quantification and analysis

Analysis of the in vitro electrophysiology data was performed by built-in and custom-written MATLAB code (MathWorks). Data collection and analysis were not performed blinded to the conditions of the experiments. Power analysis was used a priori to design experiments and determine sample sizes *n* in case of optogenetic perturbation (ArchT) experiments (for significance level of α = 0.05 and a power level of 0.80, Student’s paired *t*-test, based on the data from our previous recordings of spontaneous TRN firing and cortical LFP activity under baseline conditions). For all other experiments, no statistical methods were used to pre-determine sample sizes, but our sample sizes are similar to those reported in previous publications^55–57^.

#### Spiking data

Spikes were detected by Spike2 7.0 software. Data were downsampled to 10,000 Hz. We defined bursts as action potential packages with inter-spike intervals (ISIs) below 10 ms. aIBF was calculated as the average of the reciprocal of all ISIs within the burst. Responses of TRN cells to L5 stimulation were defined as single spikes within 30 ms or bursts with the first spikes within 30 ms after the start of the laser pulse.

#### Cortical LFP data

LFP data were downsampled to 10,000 Hz, except for the FFT analysis, where cortical LFP was downsampled to 500 Hz. For the FFT analysis, Welch’s power spectral density estimate was used. LFP traces were Z-scored. Wavelet calculations were performed using Morlet wavelet transform.

#### STA analysis

STAs were calculated as the averages of LFP traces ± 100 ms around the TRN single spikes or around the first spikes of the TRN bursts, except for Supplementary Fig. 8, where all spikes were treated individually. TRN spiking events were selected into five categories: single spikes and four burst categories according to the aIBFs. Boundaries of the four categories were defined by the 25th, 50th and 75th percentiles. STA peak amplitude was calculated for the fourth burst category (hf bursts). Because STA peaks for the first burst category (lf bursts) were often not well-defined, we calculated the STA amplitude for lf bursts at the position of the hf STA peak. For calculating the average lags of the LFP peaks from *t* = 0 ms, peak amplitudes were calculated for individual LFP events, and their lags from 0 were averaged.

#### Correlation between cortical LFP slope and TRN burst properties

Instantaneous LFP slope for each burst was calculated as the steepness of the linear fit to a 30-ms section of the LFP data around the first spikes. TRN neurons fired at different phases of the LFP fast events. To compensate for this, and to measure the steepness of the positive slope of the instantaneous LFP events, we chose the 30-ms LFP sections for the linear fit as the following:

$${{{\mathrm{X}}}}_0 = {{{\mathrm{t}}}}_{{{{\mathrm{spike}}}}} - \left( {25 - {{{\mathrm{d}}}}} \right)$$

$${{{\mathrm{X}}}}_1 = {{{\mathrm{t}}}}_{{{{\mathrm{spike}}}}}\left( {5 + {{{\mathrm{d}}}}} \right).$$

where X_0_ is the start, and X_1_ is the end of the selected LFP section in ms. *t*_spike_ is the timepoint for the first spike of the burst in ms, and *d* is the lag of the STA (LFP average for all bursts) peak in ms.

We defined the Pearson correlation coefficient (*R*) and the *P* values for the correlation between the aIBFs and the slope of the instantaneous cortical LFP slope. To compare *P* values, for the optogenetic perturbation experiments, equal numbers of bursts were selected from the baseline and the ArchT recordings. Bursts were selected uniformly at random without replacement, and *R* and *P* values were calculated. This was repeated 200 times, and the averages of *R* and *P* values were calculated.

#### Fast LFP event detection

Next, 30-ms-long sections of the (120–300-second-long) LFP recordings were randomly selected 200,000 times, and the steepness of the linear fits for these sections was calculated. The steepest LFP sections with steepness values larger than the 98th percentile were selected. Then, we searched for the peaks of these fast LFP events. Maximum amplitudes in a 100-ms time window around the selected LFP sections were detected. Peaks with maximum amplitudes larger than the LFP mean ± 3 s.d. were defined as fast event peaks.

#### Modulation of firing rate by cortical fast events

Firing rate of neurons ± 200 ms around the peaks were calculated in 10-ms bins. Firing rate averages for bins ± 50 ms around the peaks were compared to the averages for all the other bins (baseline). Neurons were defined as modulated if the average firing rate around the peaks was larger than the mean ± 2 s.d. of the baseline firing rate.

#### Quantification of L5 boutons in the TRN

For investigating L5 bouton distribution in the TRN, Thy1-ChR2-EYFP mice (*n* = 3) were used. After perfusion, the brain was sliced into 50-µm-thick coronal slices. To define the boundaries of the TRN, PV staining was carried out (see the Methods ‘Histology’ section). From each mouse, every fifth slice containing the TRN was used, from which three (70 µm × 70 µm × 12 µm) confocal z-stacks were taken, with a 0.12-µm step size (Olympus confocal microscope). The pictures were deconvolved with Xming software. Stacks were manually sorted into three categories: not boutons, 1–5 boutons (sparse innervation) and 5+ boutons (dense innervation). Cortical origin of the boutons was shown by Vglut1 staining and lack of Vglut2 staining.

#### Box plots

The box shows the first to the third quartiles with a line at the second quartile. Whisker ends label the minimum and maximum values. Average is marked by x. Values 1.5 times the interquartile range larger than the third quartile or 1.5 times the interquartile range smaller than the first quartile were considered as outliers. Outiliers were included in the analysis.

#### Statistics

Statistical comparisons were performed using the Mann–Whitney *U*-test for unpaired sets of data and the Student’s paired *t*-test for paired sets of data with normal distribution (tested by one-sample Kolmogorov–Smirnov test). Correlation between two sets of data was measured using Pearson correlation. For testing relationship between categorical variables, the chi-square statistic was used. Statistical significance was set at 0.05. Normality and equal variances were formally tested. Significance levels are indicated as follows: *P < 0.05; **P < 0.01; ***P < 0.001; NS, not significant. Results are given as mean ± s.e.m.

### Mouse Light Neuron Browser

For testing the presence of L5–TRN collaterals, we analyzed single-cell reconstruction data of the Mouse Light Neuron Browser^29^ (https://www.janelia.org/open-science/mouselight-neuronbrowser). L5 cortical neurons projecting to the thalamus were involved in the analysis. L5 neurons not targeting the thalamus were found to avoid the TRN and were not involved. For determining the soma position, we mapped the raw sample data and specified anatomical categories to match the nomenclature of our tracing experiments (rostral and caudal boundaries in M2 and mPFC were defined at bregma 1.8 mm). We used the raw images to validate the presence of L5–TRN collaterals.

### Reporting summary

Further information on research design is available in the Nature Portfolio Reporting Summary linked to this article.

## Online content

Any methods, additional references, Nature Portfolio reporting summaries, source data, extended data, supplementary information, acknowledgements, peer review information; details of author contributions and competing interests; and statements of data and code availability are available at 10.1038/s41593-022-01217-z.

## Supplementary information

Supplementary Information Supplementary Table 1

Reporting Summary

## Data Availability

Individual data points used to create the figures are available in figshare (10.6084/m9.figshare.21395547). All raw data that support the findings, tools and reagents will be shared on an unrestricted basis; requests should be directed to the corresponding authors. Concerning the data, we are able to provide the following datasets upon reasonable request: confocal images of the full extent of the injection sites; terminal arbors; dendritic and axonal processes of individual TRN cells; raw Spike2 files of individual TRN cell and cortical LFP activities; serial EM images of L5 and L6 axon terminals in TRN; and raw in vitro data.

## Code availability

Custom codes used in the paper are available at https://github.com/hadingernora/Hadinger-et-al.-2022-NN-codes.
