# Supplementary material for: Region-selective control of the thalamic reticular nucleus via cortical layer 5 pyramidal cells
Source: Nat Neurosci. 2022 Dec 22;26(1):116–30. doi: 10.1038/s41593-022-01217-z (PMC9829539; doi:10.1038/s41593-022-01217-z)
Supplement: Supplementary file 1 — Supplementary Table 1 [file 41593_2022_1217_MOESM1_ESM.pdf]

nature neuroscience

Article

https://doi.org/10.1038/s41593-022-01217-z

Region-selective control of the thalamic reticular nucleus via cortical layer 5 pyramidal cells

In the format provided by the authors and unedited

Frontal cortex			
DOI	Soma position	TRN collateral	Thalamic target
AA0011	M2 rostral	+	VM, IL, MD, Re
AA0114	M2 rostral	+	VM, IL, Sub
AA0115	M1	+	VM, IL, Pf, MD, Sub
AA0119	Orbitofrontal	+	Re, Pf
AA0121	Orbitofrontal	-	Sub, Re, MD, Pf
AA0122	M2 rostral	+	VM, IL, Pf, MD
AA0131	M1	-	VM
AA0132	M1	-	IL
AA0135	M1	-	Po
AA0179	M2 caudal	+	Re, IL
AA0181	M1	+	VM, MD, Pf, VA/VL
AA0182	M2 rostral	-	VM, IL, Pf, Po
AA0245	M2 rostral	+	VM, IL, Pf, MD, Re
AA0250	M2 rostral	+	VM, IL,VA/VL
AA0261	M2 caudal	+	VM, MD, IL, Pf, Sub
AA0415	M2 rostral	+	VM, Pf, Po
AA0576	M2 rostral	-	MD, IL
AA0583	M2 caudal	+	VM, IL, Re, VA/VL, Po
AA0587	M1	+	VM, IL, Pf, VA/VL, Po
AA0617	M1	+	IL, Pf, Po
AA0644	M1	-	VM, Sub, Re, VA/VL, VPM, Po
AA0726	M2 caudal	+	VM, MD, IL, Pf, Sub
AA0764	M2 caudal	-	AM, MD, Re, VA/VL
AA0772	M2 rostral	+	VM, IL, Pf, VA/VL
AA0780	M2 rostral	-	VM, MD, IL, Pf, IMD
AA0788	M2 rostral	+	VM, IL, Pf
AA0791	M2 caudal	-	IL, Pf, LD
AA0792	M2 caudal	+	VM, MD, IL, Pf, VA/VL
AA0794	M2 caudal	+	VM, MD, IL, Pf, VA/VL
AA0796	mPFC caudal	+	VM, MD, Re, AM,VA/VL
AA0926	M2 caudal	+	IL, Re, Po
AA0927	M1	+	IL, Po
AA1050	M1	+	VA/VL
AA1051	M1	+	VM, IL, Pf
AA1537	M2 caudal	+	LP
AA1540	M1	-	VM, Sub
AA1543	M1	-	VM
AA1544	M2 caudal	-	VM, Pf
Sensory cortices			
AA0001	S1	+	Po
AA0919	Aud	-	LP
AA0941	Aud	-	LP
AA0944	S1	-	Po
AA0945	S1	-	Po, IL, Re, LD
AA0949	Vis	-	LD
AA0956	S1	-	Po
AA1049	S1	-	Po, VPL
AA1182	Vis	-	LD, vLGN
AA1248	Vis	-	VLG

Supplementary table 1
