# Supplementary material for: Region-selective control of the thalamic reticular nucleus via cortical layer 5 pyramidal cells
Source: Nat Neurosci. 2022 Dec 22;26(1):116–30. doi: 10.1038/s41593-022-01217-z (PMC9829539; doi:10.1038/s41593-022-01217-z)
Supplement: Supplementary file 2 — Reporting Summary [file 41593_2022_1217_MOESM2_ESM.pdf]

Corresponding author(s): László Acsády, Nóra Hádinger

Last updated by author(s): Oct 25, 2022

Reporting Summary

Nature Portfolio wishes to improve the reproducibility of the work that we publish. This form provides structure for consistency and transparency in reporting. For further information on Nature Portfolio policies, see our Editorial Policies and the Editorial Policy Checklist.

Statistics

For all statistical analyses, confirm that the following items are present in the figure legend, table legend, main text, or Methods section.

n/a	Confirmed	
☐	☒	The exact sample size (*n*) for each experimental group/condition, given as a discrete number and unit of measurement
☐	☒	A statement on whether measurements were taken from distinct samples or whether the same sample was measured repeatedly
☐	☒	The statistical test(s) used AND whether they are one- or two-sided<br>*Only common tests should be described solely by name; describe more complex techniques in the Methods section.*
☐	☒	A description of all covariates tested
☐	☒	A description of any assumptions or corrections, such as tests of normality and adjustment for multiple comparisons
☐	☒	A full description of the statistical parameters including central tendency (e.g. means) or other basic estimates (e.g. regression coefficient) AND variation (e.g. standard deviation) or associated estimates of uncertainty (e.g. confidence intervals)
☐	☒	For null hypothesis testing, the test statistic (e.g. *F*, *t*, *r*) with confidence intervals, effect sizes, degrees of freedom and *P* value noted<br>*Give P values as exact values whenever suitable.*
☒	☐	For Bayesian analysis, information on the choice of priors and Markov chain Monte Carlo settings
☒	☐	For hierarchical and complex designs, identification of the appropriate level for tests and full reporting of outcomes
☐	☒	Estimates of effect sizes (e.g. Cohen's *d*, Pearson's *r*), indicating how they were calculated

Our web collection on statistics for biologists contains articles on many of the points above.

Software and code

Policy information about availability of computer code

Data collection	Spike2 5.0 was used for recording neural signals.<br>Megaview software was used for taking serial electromicrographs.<br>NIS-Elements AR and Olympus FluoView™ softwares were used for taking confocal images.
Data analysis	For the analysis of in vivo electrophysiology data custom and built in codes in Spike2 7.0 and MatlabR2016b were used as described in Methods.<br>For analyzing and 3D-reconstructing electron microscopy data, ImageJ 1.52i (Fiji) and Reconstruct™ softwares were used respectively.<br>Xming software was used for confocal image deconvolution.

For manuscripts utilizing custom algorithms or software that are central to the research but not yet described in published literature, software must be made available to editors and reviewers. We strongly encourage code deposition in a community repository (e.g. GitHub). See the Nature Portfolio guidelines for submitting code & software for further information.

Data

Policy information about availability of data

All manuscripts must include a data availability statement. This statement should provide the following information, where applicable:

- Accession codes, unique identifiers, or web links for publicly available datasets
- A description of any restrictions on data availability
- For clinical datasets or third party data, please ensure that the statement adheres to our policy

Mouse Light Neuron Browser dataset is available at https://www.janelia.org/open-science/mouselight-neuronbrowser. Individual data points used to create the figures are available as Source Data Files. All raw data that support the findings, tools and reagents will be shared on an unrestricted basis; requests should be directed to the corresponding authors. Concerning the data we are able to provide the following datasets upon request: Confocal images of the full extent of the injection sites, terminal arbors, dendritic and axonal processes of individual TRN cells, raw Spike2 files of individual TRN cell and cortical LFP activities, serial EM images of L5 and L6 axon terminals in TRN and raw in vitro data. Custom codes will be available in a repository (link provided in proofs).

Field-specific reporting

Please select the one below that is the best fit for your research. If you are not sure, read the appropriate sections before making your selection.

☒ Life sciences ☐ Behavioural & social sciences ☐ Ecological, evolutionary & environmental sciences

For a reference copy of the document with all sections, see nature.com/documents/nr-reporting-summary-flat.pdf

Life sciences study design

All studies must disclose on these points even when the disclosure is negative.

Sample size	To predetermine sample sizes, either we acted in accordance with our previous practice (Bokor et al, 2005; Barthó et al, 2007; Bodor et al, 2008), or in case of the optogenetic perturbation (ArchT) experiments, minimum sample sizes were calculated via power analysis based on the data from our previous recordings of spontaneous TRN firing and cortical LFP activity.
Data exclusions	No data was excluded from the analyis. Criteria for data collection for each experiments are described in the Results or Methods section.
Replication	All experiments were replicated in multiple animals. The sample size, and the number of animals for each experiment is included in the main text, figure legends or supplementary figure legends. For electrophysiological perturbation experiments multiple controls were included in the experimental design. To report statistics we used whisker plots and for electropysiological perturbation experiments, individual datapoints were shown demonstrating that the effects are representative and not caused by a small fraction of the sample.
Randomization	All subjects were randomly assigned to groups.
Blinding	The experimenters were not blind to the conditions of the animals. All physiology data were processed by automated software, eliminating the possibility of biases in data processing.

Reporting for specific materials, systems and methods

We require information from authors about some types of materials, experimental systems and methods used in many studies. Here, indicate whether each material, system or method listed is relevant to your study. If you are not sure if a list item applies to your research, read the appropriate section before selecting a response.

Materials & experimental systems

n/a	Involved in the study	
☐	☒	Antibodies
☒	☐	Eukaryotic cell lines
☒	☐	Palaeontology and archaeology
☐	☒	Animals and other organisms
☒	☐	Human research participants
☒	☐	Clinical data
☒	☐	Dual use research of concern

Methods

n/a	Involved in the study	
☒	☐	ChIP-seq
☒	☐	Flow cytometry
☒	☐	MRI-based neuroimaging

Antibodies

Antibodies used	Primary antibodies:<br>Rabbit polyclonal anti-VGLUT2 (Synaptic systems) #135404, 1:10000

Rabbit polyclonal anti-Calbindin (Swant: CB-38a), 1:2000
(https://www.swant.com/pdfs/Rabbit_anti_calbindin_D-28k_CB38.pdf)

Rabbit polyclonal anti-Fluorogold (Millipore: AB153-I), 1:10000
https://www.merckmillipore.com/HU/hu/product/Anti-Fluorescent-Gold-Antibody,MM_NF-AB153-I?ReferrerURL=https%3A%2F%2Fwww.google.com%2F&bd=1

Chicken polyclonal anti-GFP (ThermoFisher Scientific: A10262), 1:5000
(https://www.thermofisher.com/antibody/product/GFP-Antibody-Polyclonal/A10262)

Rabbit polyclonal anti-GFP (ThermoFisher Scientific: A11122), 1:2000
(https://www.thermofisher.com/antibody/product/GFP-Antibody-Polyclonal/A-11122)

Rabbit polyclonal anti-mCherry (BioVision: 5993-100), 1:3000
(https://www.biovision.com/documentation/datasheets/5993.pdf)

Mouse monoclonal anti-Parvalbumin (Sigma: PARV-19), 1:2000
https://www.sigmaaldrich.com/specification-sheets/158/550/P3088-BULK________SIGMA____.pdf

Rabbit polyclonal anti-Vglut1 (Millipore: ABN1647), 1:10000
https://www.merckmillipore.com/HU/hu/product/Anti-VGluT1,MM_NF-ABN1647?bd=1#anchor_COA

Secondary antibodies:

Alexa488 conjugated Goat anti-Chicken antibody (ThermoFisher: A-11039), 1:500

Cy3 conjugated AffiniPure Donkey anti-Rabbit IgG (Jackson ImmunoResearch: AB_2307443), 1:500

Cy5 conjugated AffiniPure Donkey anti-Mouse IgG (Jackson ImmunoResearch: AB_2340820), 1:500
Cy5 conjugated AffiniPure Donkey anti-Rabbit IgG (Jackson ImmunoResearch: AB_2340607), 1:500

Biotinylated goat anti-rabbit – bGAR (Vector Laboratories: BA-1000), 1:300
Biotin-SP (long spacer) AffiniPure Donkey Anti-Rabbit IgG (Jackson ImmunoResearch: AB_2340593), 1:300

Validation

The specificity of the primary antibodies was validated by the manufacturer. For details, see the manufacturer's website.

Animals and other organisms

Policy information about studies involving animals; ARRIVE guidelines recommended for reporting animal research

Laboratory animals

Adult (2-5 months old) male mice were used for all experiments.
Rbp4-Cre line: Mutant Mouse Regional Resource Center (MMRRC_031125-UCD)
Thy1-ChR2-YFP line: The Jackson Laboratory (#007612)
Thy1-Cre line: The Jackson Laboratory (#006143)
Ntsr1-Cre line: Mutant Mouse Regional Resource Center (MMRRC_030780-UCD)

Wild animals

This study did not involve wild animals.

Field-collected samples

This study did not involve samples collected from the field.

Ethics oversight

All animal use was approved by the Animal Welfare Committee of the Institute of Experimental Medicine, Budapest, in accordance with the regulations of the European Community's Council Directive of November 24, 1986 (86/609/EEC). The experiments were approved by the National Animal Research Authorities of Hungary (PE/EA/877-7/2020).

Note that full information on the approval of the study protocol must also be provided in the manuscript.
